# From Flora to Pharmaceuticals: 100 new additions to angiosperms of Gafargaon subdistrict in Bangladesh and unraveling antidiabetic drug candidates targeting DPP4 through *in silico* approach

**DOI:** 10.1371/journal.pone.0301348

**Published:** 2024-03-29

**Authors:** Sheikh Sunzid Ahmed, M. Oliur Rahman

**Affiliations:** Department of Botany, Faculty of Biological Sciences, University of Dhaka, Dhaka, Bangladesh; Bangladesh Agricultural University, BANGLADESH

## Abstract

Addition to the angiosperm flora provides essential insights into the biodiversity of a region, contributing to ecological understanding and conservation planning. Gafargaon subdistrict under Mymensingh district in Bangladesh represents a diverse population of angiosperms with a multifaceted ecosystem that demands re-evaluation of the existing angiosperm diversity of Gafargaon to update the status of angiosperm taxa and facilitate their conservation efforts. With this endeavor, a total of 100 angiosperm taxa belonging to 90 genera and 46 families were uncovered as additional occurrence in Gafargaon. The species in the area showcased a variety of life forms, including 63 herbs, 14 shrubs, 14 trees, and 9 climbers. Among the recorded taxa, *Chamaecostus cuspidatus* (Nees & Mart.) C.D. Specht & D.W. Stev. was selected for antidiabetic drug design endeavor based on citation frequency and ethnomedicinal evidence. A total of 41 phytochemicals of *C*. *cuspidatus* were screened virtually, targeting the Dipeptidyl peptidase 4 protein through structure-based drug design approach, which unveiled two lead compounds, such as Tigogenin (-9.0 kcal/mol) and Diosgenin (-8.5 kcal/mol). The lead candidates demonstrated favorable pharmacokinetic and pharmacodynamic properties with no major side effects. Molecular dynamics simulation revealed notable stability and structural compactness of the lead compounds. Principal component analysis and Gibbs free energy landscape further supported the results of molecular dynamics simulation. Molecular mechanics-based MM/GBSA approach unraveled higher free binding energies of Diosgenin (-47.36 kcal/mol) and Tigogenin (-46.70 kcal/mol) over Alogliptin (-46.32 kcal/mol). The outcome of the present investigation would enrich angiosperm flora of Gafargaon and shed light on the role of *C*. *cuspidatus* to develop novel antidiabetic therapeutics to combat diabetes.

## 1. Introduction

Floristics, the comprehensive study of plant species within a specific region, is fundamental to advancing our current understanding of biodiversity and serves as a cornerstone in conservation biology [1]. The continual expansion of a flora’s checklist through the addition of new taxa holds significant promise in providing essential baseline data that not only enhances our comprehension of ecosystem dynamics but also aids in the formulation of effective conservation strategies [2]. The inclusion of new taxa provides insights into the adaptive capacities of species, contributes to the refinement of knowledge about the distribution and abundance of plant life, and further enables the identification of key areas for conservation intervention [3, 4]. Gafargaon subdistrict under Mymensingh district in Bangladesh is located in 24°15’ to 24°33’N and 90°27’ to 90°39’E, spanning an area of 401.16 sq. km. Diverse ecosystems of Gafargaon contribute significantly to the local economy, environment, and primary healthcare system by supporting dense populations of angiosperms [5, 6]. Regrettably, over the years, human activities have detrimentally impacted the ecosystems of Gafargaon, resulting in the decline of various plant species, some of which are now rare and endangered. Rahman et al. (2019) conducted an investigation on Gafargaon flora and documented 203 taxa belonging to 174 genera and 75 families [5]. The study identified several threats, such as habitat degradation, encroachment, over-exploitation of medicinal plants, exotic plantation etc. These challenges necessitate a thorough reassessment of the existing angiosperm diversity of Gafargaon to gauge the status of angiosperm taxa, streamline their preservation efforts and safeguard existing plant species of Gafargaon.

Floristics is instrumental in uncovering medicinal species across diverse plant groups by examining plant diversity and grasping the interconnections among different plant species. Precise species identification becomes imperative before revealing potential drug candidates sourced from plants, and this essential identification process is facilitated by comprehensive taxonomic studies [7]. The wealth of plant biodiversity cataloged through floristics, therefore, may serve as a foundation for sourcing potential drug candidates, bridging the gap between traditional botanical knowledge and cutting-edge pharmaceutical research. This interdisciplinary approach harnesses the traditional knowledge encoded in floristics to inform and direct the Structure Based Drug Design (SBDD) strategies, fostering a more comprehensive and sustainable exploration of medicinal resources within natural plant ecosystems [8].

SBDD represents a contemporary computational biology-driven paradigm transforming the drug discovery landscape by expediting processes, curtailing expenses, and expanding research capabilities [9–13]. The convergence of floristics and SBDD helps in guiding the progression of antidiabetic drug development. Diabetes is an escalating global concern, affecting approximately 422 million people worldwide and directly contributing to 1.5 million deaths annually. The rising prevalence of diabetes underscores the urgent need for innovative strategies to address this pervasive health issue [https://www.who.int]. The Dipeptidyl peptidase 4 (DPP4) enzyme is a key player involved in the regulation of glucose homeostasis [14] and plays a critical role in degrading incretin hormones that stimulate insulin secretion. Inhibiting DPP4 can enhance incretin activity, leading to improved glycemic control in individuals with diabetes [15]. Several SBDD studies have used this enzyme as the target protein to propose some bioactive phytochemicals as antidiabetic drug candidates from various angiosperms, such as *Pueraria tuberosa*, *Moringa oleifera*, *Ocimum tenuiflorum* and *Amberboa ramosa* [16–19]. However, no studies have been reported *in silico* to denote the antidiabetic potential of *Chamaecostus cuspidatus* targeting the DPP4 protein. Hence, this ethnomedicinal plant species from our floristics investigation was selected to design novel drug candidates against diabetes.

Phytochemicals, sourced from plants, are instrumental in drug development, as evidenced by FDA-approved medications harnessing their therapeutic properties. Digoxin, extracted from *Digitalis purpurea*, effectively manages congestive heart failure, while Artemisinin from *Artemisia annua* tackles malaria, notably lowering mortality rates. Moreover, *Taxus brevifolia* phytochemicals form the basis of Paclitaxel, a vital chemotherapy agent for diverse cancers. Metformin, a widely prescribed drug for type 2 diabetes, is derived from *Galega officinalis*, exemplifying the utilization of plant-based compounds in modern medicine. These instances underscore phytochemicals’ significant contribution to modern medicine, providing potent remedies for critical illnesses [20, 21].

The present investigation sought to revisit the angiosperm diversity within Gafargaon subdistrict, with a specific focus on identifying previously undiscovered taxa, particularly those with medicinal significance. Additionally, the objective was to put forth potential antidiabetic drug candidates derived from the selected medicinal species. To achieve this, a Structure Based Drug Design (SBDD) approach was employed, targeting the DPP4 protein. This dual-purpose investigation aimed not only to contribute to the current understanding of the angiosperm diversity in Gafargaon region but also to explore novel avenues for developing antidiabetic medications based on the identified medicinal plant.

## 2. Materials and methods

### 2.1 Taxonomic inventory

A comprehensive taxonomic survey was conducted in the Gafargaon upazila of Mymensingh district over the course of 25 botanical expeditions, spanning all seasons from July 2020 to June 2023. During these expeditions, plant specimens bearing flowers and/or fruits were collected, thoroughly examined, and preserved in accordance with established herbarium protocol [22, 23]. We identified the collected specimens by consulting standard botanical references and by cross-referencing with pre-identified specimens housed at the Dhaka University Salar Khan Herbarium (DUSH) [5, 24–29]. Nomenclature for each taxon was updated in accordance with the most recent literature and online databases, including World Flora Online and International Plant Names Index. The plant families were arranged in alignment with the APG IV system of classification [30]. Within each family, the genera and species were arranged in alphabetical order. Each species was accompanied by its updated nomenclature, growth habit, brief description, habitat, flowering and fruiting period (1–12, indicating months), usage, and voucher number. Information on the medicinal uses of these plants was gathered through interviews with local residents. Voucher specimens were meticulously preserved at the DUSH.

### 2.2 Drug design endeavor

Based on field observation, ethnomedicinal importance, consent of local people and novelty, *Chamaecostus cuspidatus* (Nees & Mart.) C.D. Specht & D.W. Stev. was selected for designing antidiabetic drug candidates with its bioactive phytocompounds. A comprehensive structure-based drug design (SBDD) protocol was employed *in silico* to explore antidiabetic potential of this medicinal herb.

#### 2.2.1 Target protein preparation

The promising target Dipeptidyl peptidase 4 (DPP4) was selected as the macromolecular receptor and fetched from the Protein Data Bank with PDB ID “2ONC” [31]. DPP4 has been chosen for its broad relevance in the field of *in silico* drug design research, due to its pivotal role in the degradation of incretin hormones, which are known to stimulate the secretion of insulin [15]. The protein was modified with MGL-AutoDockTools v.1.5.6 and energy minimized using SWISS-PDB viewer v4.10 [32, 33].

#### 2.2.2 Ligands preparation

*C*. *cuspidatus*, commonly referred to as the ’Insulin Plant,’ is highly esteemed in traditional medicine for its efficacy in managing diabetes. This botanical gem boasts a plethora of bioactive phytochemicals, presenting a promising avenue for the discovery and development of novel antidiabetic drug candidates. Forty-one compounds of *C*. *cuspidatus* were downloaded from the PubChem database (https://pubchem.ncbi.nlm.nih.gov) after extensive literature survey [34–36]. Alogliptin, a promising DPP4 inhibitor was used as the control drug and fetched from PubChem [37]. All the ligands went through energy minimization process in Open Babel v.2.3.1 and later converted to PDBQT before molecular docking process [38, 39].

#### 2.2.3 Active site identification

The target protein was subjected to active site prediction to conduct a site-specific docking. PrankWeb server was utilized for determining active site in default settings [40]. The prediction was made by focusing on points positioned on the solvent-accessible surface of proteins. The PDB file of the target protein was uploaded as a custom structure in the PrankWeb server, with the conservation box checked. After generating output, pocket rank, pocket score, confidence level and conservation scores of various output options were analyzed and compared to determine the optimal selection.

#### 2.2.4 Site-specific molecular docking

Prior to conducting site-specific docking, a grid box was generated using AutoDockTools v.1.5.6 incorporating the binding site residues predicted by the PrankWeb server. The size coordinates in the grid box were 54 × 56 × 62, and the center coordinates were 45.589 × -17.354 × -31.654. The grid box covered the active sites of the DPP4 protein. Subsequently, molecular docking analysis was performed with AutoDock Vina [41]. The resulting docked complexes were analyzed for molecular interactions via PyMol and BIOVIA Discovery Studio Visualizer v21.1.0.20298 [42].

#### 2.2.5 Drug-likeness evaluation via ADMET

ADMET (Absorption, Distribution, Metabolism, Excretion, and Toxicity) characteristics play a crucial role in influencing the pharmacological effects and overall effectiveness of drug candidates, making them vital factors in the selection of lead compounds. SwissADME server was utilized to assess ADME properties of the selected phytochemicals after molecular docking analysis [43]. Subsequently, ProToxII and STopTox servers were utilized for toxicity evaluation [44, 45].

#### 2.2.6 Molecular dynamics simulation (MDS)

For MDS in GROMACS v.2020.6, ligand topology files were generated using the CgenFF server. All the systems were solvated with the TIP3P (Transferable Intermolecular Potential with 3 Points) water model, employing a triclinic box positioned 1 nm away from the protein surface [46]. Neutralization was accomplished by adding sufficient sodium and chloride ions (0.15 M salt). Energy minimization was carried out using the CHARMM36m force field with 5000 steps. In the process of system equilibration and molecular dynamics (MD) simulations, the NVT/NPT ensemble was applied, keeping the pressure and temperature at 1 bar and 300K, respectively. Particle Mesh Ewald (PME) was utilized to calculate long-range interactions. Subsequently, a 100 ns MD production run was executed, targeting approximately 1000 frames per simulation. The time integration step was set to 2fs, and the snapshot interval was configured at 100 ps [42]. For trajectory analyses, GROMACS utilities were employed, such as *gmx rms*, *gmx rmsf*, *gmx gyrate* and *gmx sasa*, which produced trajectory results formatted in CSV (Comma-Separated Values). Subsequently, all the CSV data were visualized using Microsoft Excel v.2013 to assess various parameters including Root Mean Square Deviation (RMSD), Root Mean Square Fluctuation (RMSF), Radius of Gyration (Rg), and total Solvent Accessible Surface Area (SASA). These analyses were conducted to evaluate the dynamic stability between the DPP4 receptor and potential lead candidates derived from *C*. *cuspidatus*.

#### 2.2.7 Principal component analysis

Principal Component Analysis (PCA) is a widely employed analytical method for depicting the slow and functional motions of biomolecules [47]. To obtain the principal components of the protein-ligand complexes, the eigenvalue and eigenvectors of the covariance matrix were calculated and diagonalized. The eigenvectors indicate the direction of motion, while the eigenvalues illustrate both the direction and magnitude of motion. The covariance matrix for PCA was computed for backbone C alpha atoms using the GROMACS analysis tool, *gmx covar*, which both constructs and diagonalizes the covariance matrix. Additionally, another GROMACS pre-built tool, *gmx anaeig*, was utilized to assess the overlap between principal components and trajectory coordinates.

#### 2.2.8 Gibbs free energy landscape analysis

The free energy landscape (FEL) serves as a representation of potential conformations assumed by a protein during a molecular dynamics simulation, incorporating Gibbs free energy. The FEL elucidates two variables that capture specific system properties and assess conformational variability. It was generated using the probability distribution derived from the essential plane formed by the first two eigenvectors. The construction of the FEL was carried out using the *gmx sham* tool. Afterwards, two Python scripts were employed to visualize the results and produce 2D and 3D images. The Python scripts can be found in the S1 File.

#### 2.2.9 MM/GBSA free binding energy

The Prime package of the Schrödinger v.2020-3 software was utilized for MM/GBSA (Molecular Mechanics/Generalized Born Surface Area) calculations [48]. The OPLS2005 force field and VSGB continuum solvation model were selected to estimate the free binding energies [49] utilizing the formula:

ΔG(bind)=ΔG(solv)+ΔE(MM)+ΔG(SA) (1)

Where, ΔG(solv) denotes variation in GBSA solvation energy between protein-inhibitor complex and the total solvation energies of the unbound inhibitor and protein; ΔE(MM) signifies the discrepancy in minimized energies between the protein-inhibitor complex and total energies of unbound inhibitor and protein; ΔG(SA) represents variation in surface area energies of the protein-inhibitor complex and the aggregated surface area energies of the individual components.

#### 2.2.10 Structural analogs and drug target class

Drug target class was predicted using SwissTargetPrediction server [50]. Canonical SMILES was uploaded in the server checking *Homo sapiens* datasets. Structurally similar analogs were predicted using SwissSimilarity server [51]. In target class prediction, the *Homo sapiens* dataset is prioritized due to its exclusive focus on human proteins. This selection is crucial, especially in pharmaceutical applications, as it ensures that the predicted drug targets are pertinent to human biology. By employing the *Homo sapiens* dataset, the predictions are customized to human-specific targets, thus enhancing the probability of identifying drug candidates with therapeutic significance in humans. This approach not only increases the likelihood of discovering effective and safe drugs for human use but also ensures that the predictions remain clinically relevant and aligned with human biology.

## 3. Results

### 3.1 Taxonomic inventory and enumeration

**Fig 1:**
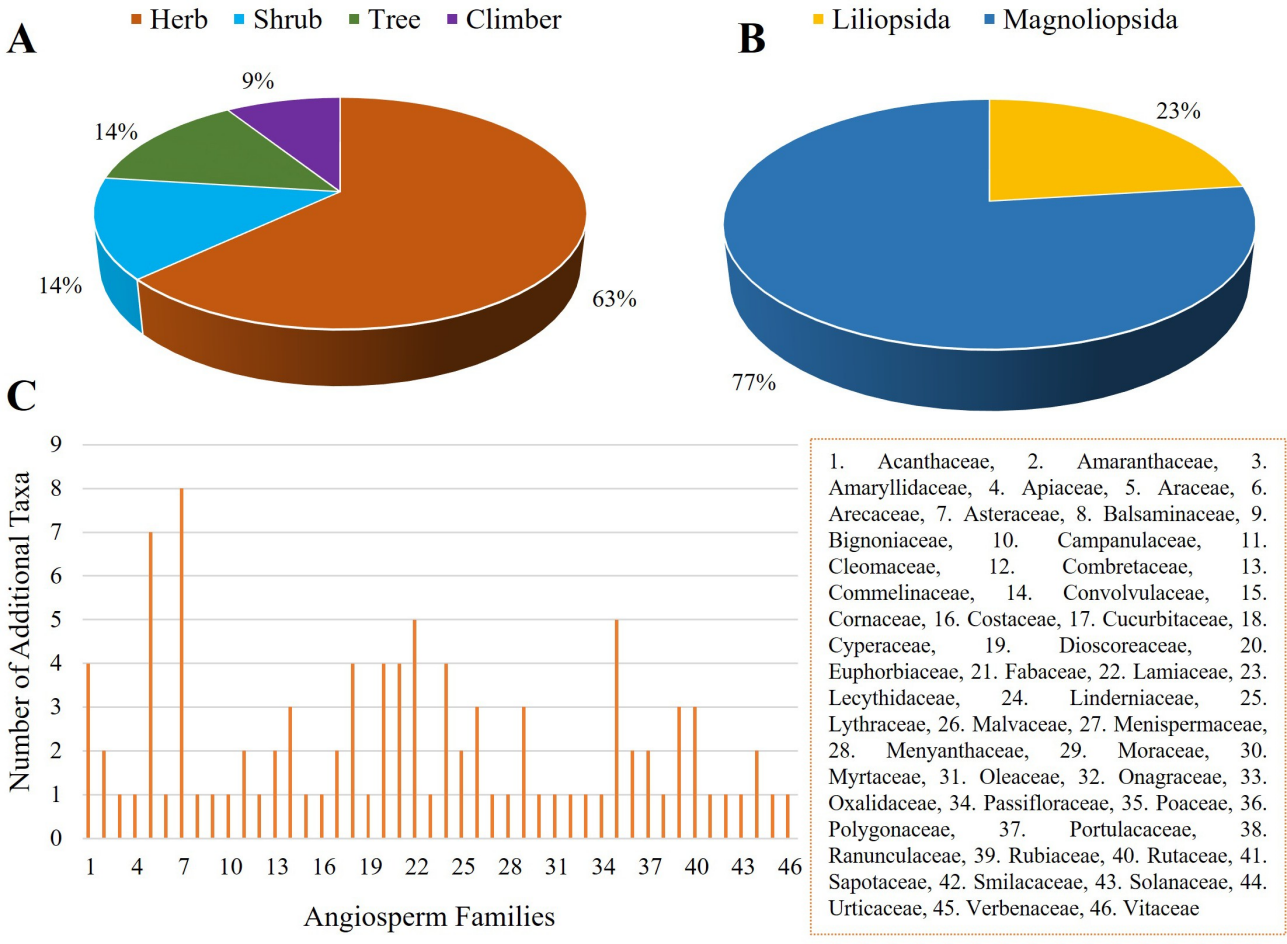
Overview of the additional angiosperm taxa in Gafargaon subdistrict. A. Habit-wise categorization of the recorded taxa. B. Class-wise categorization showing the percentage of the Liliopsida and Magnoliopsida species. C. Number of additional taxa with representative families of angiosperms.

**Fig 2:**
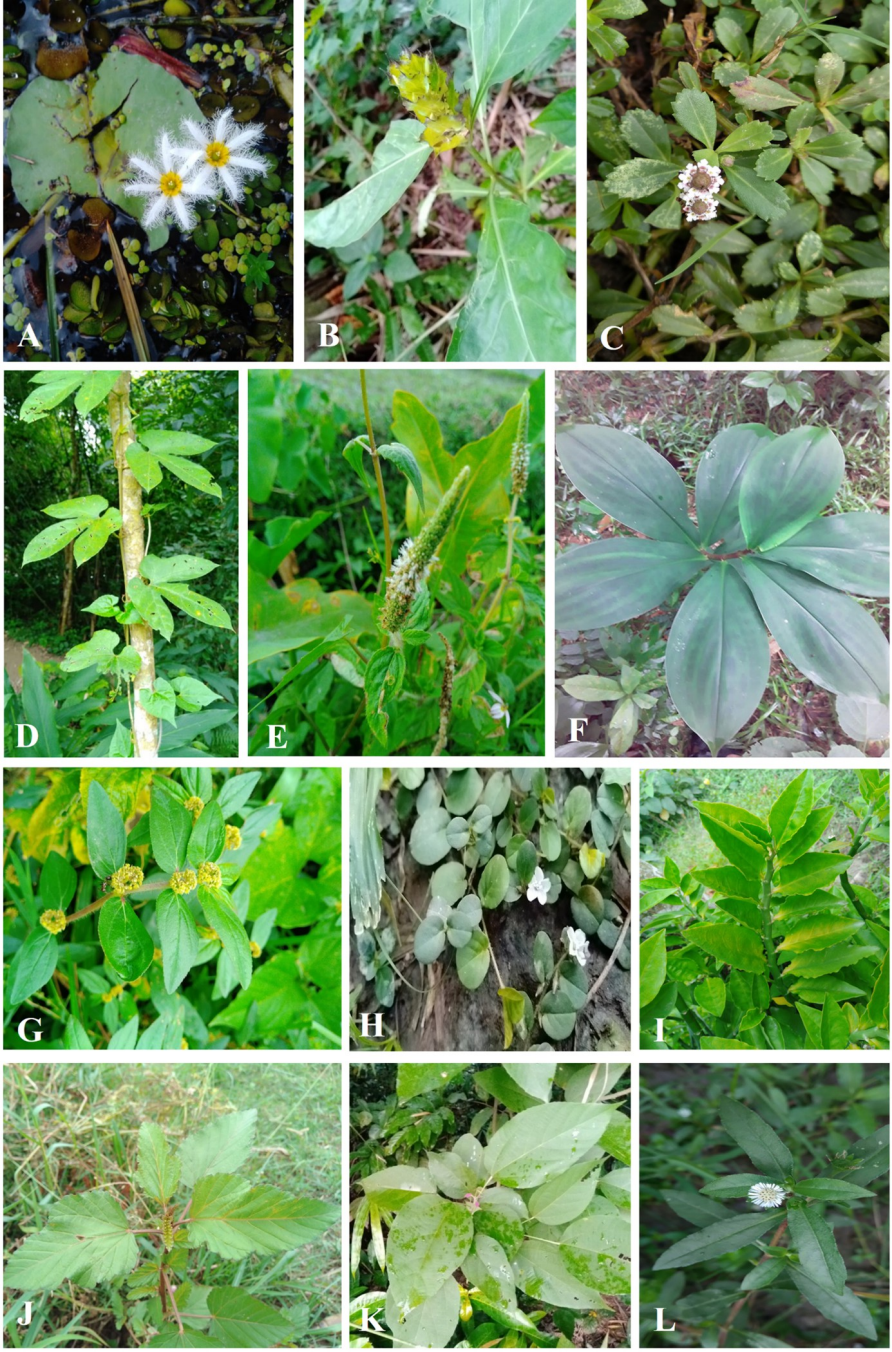
Some taxa with ethnomedicinal uses as reported by the local inhabitants of Gafargaon. A. *Nymphoides indica*, B. *Ecbolium ligustrinum*, C. *Phyla nodiflora*, D. *Adenia trilobata*, E. *Pogostemon auricularius*, F. *Chamaecostus cuspidatus*, G. *Euphorbia hirta*, H. *Evolvulus nummularius*, I. *Euphorbia tithymaloides*, J. *Melochia corchorifolia*, K. *Oroxylum indicum*, L. *Eclipta prostrata*.

The current study unveiled the occurrence of an additional 100 species of angiosperms belonging to 90 genera and 46 families within the study area. Among these species, Liliopsida (Monocots) accounted for 23%, while Magnoliopsida (Dicots) constituted 77% of the total. The majority of the species were herbs (63%), followed by shrubs (14%), trees (14%) and climbers (9%) (Fig 1). Within the identified taxa, some ethnomedicinally important species were showcased in Fig 2.

#### Family: Araceae

**1. *Epipremnum aureum*** (Linden & André) G.S. Bunting, Ann. Missouri Bot. Gard. 50: 28 (1964). *Pothos aureus* Linden & André, Ill. Hort. 27: 69 (1880). A perennial vine with cordate to ovate leaves. Inhabits tree trunks, stone walls, shady and moist areas. Fl. & Fr.: 7–9. Used to improve indoor air quality. Voucher: Sunzid 302 (DUSH).
**2. *Epipremnum pinnatum*** (L.) Engl., Pflanzenr. IV: 60 (1908). *Pothos pinnatus* L., Sp. Pl., ed.2: 1324 (1763). A perennial climbing vine with pinnatifid leaves. Grows on tree trunks and stone walls. Fl. & Fr.: 4–5. Used to treat abscess, traumatic injury and rheumatic arthralgia. Voucher: Sunzid 303 (DUSH).
**3. *Lasia spinosa*** (L.) Thwaites, Enum. Pl. Zeyl.: 336 (1864). *Dracontium spinosum* L., Sp. Pl.: 967 (1753). A perennial, stout herb with coriaceous leaves. Inhabits roadside, scrub jungles and homestead areas. Fl. & Fr.: 1–11. Leaves and roots are used to treat intestinal diseases, piles and chronic rheumatism. Voucher: Sunzid 317 (DUSH).
**4. *Pothos chinensis*** (Raf.) Merr., J. Arnold Arbor. 29: 210 (1948). *Tapanava chinensis* Raf., Fl. Tellur. 4: 14 (1838). A large shrubby climber with ovate-oblong to lanceolate leaves. Inhabits in scrub jungles and humid areas. Fl. & Fr.: 12–6. Leaves are taken internally with *ghee* to alleviate pain. Voucher: Sunzid 341 (DUSH).
**5. *Pothos scandens*** L., Sp. Pl.: 968 (1753). *Pothos angustifolius* (Raf.) C. Presl, Epimel. Bot.:243 (1851). An epiphytic creeper with broadly winged leaves. Grows in scrub jungles, sometimes on roadsides. Fl. & Fr.: 1–12. Leaves are used to treat rheumatism. Voucher: Sunzid 342 (DUSH).
**6. *Spirodela polyrhiza*** (L.) Schleid., Linnaea 13: 392 (1839). *Lemna polyrhiza* L., Sp. Pl.: 970 (1753). A free-floating aquatic herb. Commonly observed in paddy field and lentic habitats. Fl. & Fr.: 4–10. Used as fodder, sometimes utilized to treat skin and gastro-intestinal problems. Voucher: Sunzid 350 (DUSH).
**7. *Syngonium podophyllulm*** Schott, Bot. Zeitung. 9: 85 (1851). *Pothos auritus* Willd. *ex* Schult., Mant. 3: 301 (1827). A semi-erect perennial herb. Frequently grows in moist and shady places and homestead areas. Fl. & Fr.: 5–9. Used as an ornamental plant. Voucher: Sunzid 352 (DUSH).

#### Family: Dioscoreaceae

**8. *Dioscorea alata*** L., Sp. Pl.: 1033 (1753). *Dioscorea globosa* Roxb., Fl. Ind., ed. 1832. 3: 797 (1832). A twining annual herb with ovate or deltoid-ovate leaves. Grows in well-drained fertile soils in gardens, fields and humid lowland areas. Fl. & Fr.: 10–12. Tubers are consumed internally. Decoction of tuber is used in the treatment of diabetes. Voucher: Sunzid 298 (DUSH).

#### Family: Smilacaceae

**9. *Smilax perfoliata*** Lour., Fl. Cochinch.: 622 (1790). *Smilax laurina* Kunth, Enum. Pl. 5: 248 (1850). A perennial climber with ovate to ovate-oblong leaves. Inhabits scrub jungles and village thickets. Fl. & Fr.: 11–3. Decoction of root is used to treat urinary disorders and diabetes. Voucher: Sunzid 346 (DUSH).

#### Family: Amaryllidaceae

**10. *Hymenocallis littoralis*** (Jacq.) Salisb., Trans. Hort. Soc. London 1: 338 (1812). *Pancratium littorale* Jacq., Select. Stirp. Amer. Hist.: 99 (1763). An erect herb with linear-lanceolate leaves. Grows in homestead areas and scrub jungles. Fl. & Fr.: 4–10. Flowers are used for ornamental purposes. Voucher: Sunzid 312 (DUSH).

#### Family: Arecaceae

**11. *Caryota mitis*** Lour., Fl. Cochinch.: 569 (1790). *Caryota speciosa* Linden, Ill. Hort. 28: 16 (1881). A small monoecious tree. Found in scrub jungles, sometimes in homestead areas and roadsides. Fl. & Fr.: 4–5. Used for aesthetic purposes including design of landscapes. Voucher: Sunzid 287 (DUSH).

#### Family: Commelinaceae

**12. *Murdannia nudiflora*** (L.) Brenan, Kew Bull. 7: 189 (1952). *Commelina nudiflora* L., Sp. Pl.: 41 (1753). An erect perennial herb with linear to lanceolate leaves. Grows in moist and shady homestead areas. Fl. & Fr.: 7–12. Used to treat diabetes. Voucher: Sunzid 325 (DUSH).
**13. *Tradescantia spathacea*** Sw., Prodr. Veg. Ind. Occ.: 57 (1788). *Rhoeo discolor* (L. ’Hér.) Hance, Ann. Bot. Syst. 3: 660 (1852). A stout perennial herb with lanceolate leaves. Grows in moist and shady places, gardens and homestead areas. Fl. & Fr.: 1–2. Grown as ornamental plant. Voucher: Sunzid 357 (DUSH).

#### Family: Costaceae

**14. *Chamaecostus cuspidatus*** (Nees & Mart.) C.D. Specht & D.W. Stev., Taxon 55: 158 (2006). *Costus igneus* N.E.Br., Ill. Hort. 31: 25 (1884). A perennial herb with oblong to lanceolate leaves. Found in moist, shaded areas, along riverbank and in forest undergrowth. Fl. & Fr.: 7–10. Leaves possess analgesic properties, and also used for the treatment of diabetes. Voucher: Sunzid 289 (DUSH).

#### Family: Cyperaceae

**15. *Cyperus mindorensis*** (Steud.) Huygh, Phytotaxa 166: 39 (2014). *Kyllinga mindorensis* Steud., Syn. Pl. Glumac. 2: 67 (1854). A rhizomatous perennial herb with linear leaves. Found in sunny or shaded waste land, roadsides and grassy fields. Fl. & Fr.: 6–9. Whole plant is used as fodder for cattle and horses. Voucher: Sunzid 315 (DUSH).
**16. *Cyperus rotundus*** L., Sp. Pl.: 45 (1753). *Cyperus bicolor* Vahl, Enum. Pl. Obs. 2: 340 (1805). A perennial herb with linear leaves. Grows in agricultural lands, wetlands, disturbed habitats, scrub jungles and homestead areas. Fl. & Fr.: 5–9. Tubers are used to treat nasal pain and inflammation. Voucher: Sunzid 295 (DUSH).
**17. *Fimbristylis quinquangularis*** (Vahl) Kunth, Enum. Pl. 2: 229 (1837). *Scirpus miliaceus* L., Syst. Nat. ed.10(2): 868 (1759). A perennial herb, densely tufted. Grows in agricultural lands, moist places and disturbed habitats. Fl. & Fr.: 5–11. Protects soil erosion in wetland and aquatic environments. Voucher: Sunzid 308 (DUSH).
**18. *Schoenoplectus torreyi*** (Olney) Palla, Allg. Bot. Z. Syst. 17 (Beibl.): 3 (1911). *Scirpus subterminalis* var. *cylindricus* (Torr.) T. Koyama, Canad. J. Bot. 40: 930 (1962). A perennial herb with linear leaves. Found in agricultural lands, wetlands, near ponds and homestead areas. Fl. & Fr.: 4–8. Mainly used as fodder for cattle. Voucher: Sunzid 344 (DUSH).

#### Family: Poaceae

**19. *Axonopus compressus*** (Sw.) P. Beauv., Ess. Agrostogr.: 12 (1812). *Milium compressum* Sw., Prodr. Veg. Ind. Occ.: 24 (1788). A perennial herb with linear-oblong, oblanceolate or linear-lanceolate leaves. Grows in fields, lawns and gardens, roadside verges and disturbed areas. Fl. & Fr.: 1–12. Used for the treatment of bone fracture. Voucher: Sunzid 284 (DUSH).
**20. *Eleusine indica*** (L.) Gaertn., Fruct. Sem. Pl. 1: 8 (1788). *Cynosurus indicus* L., Sp. Pl.: 72 (1753). A tufted annual or short-lived perennial herb with linear leaves. Found in dry and wet places, gardens, lawns, roadsides, rarely in scrub jungles. Fl. & Fr.: 6–8. Prevent soil erosion. Voucher: Sunzid 301 (DUSH).
**21. *Hygroryza aristata*** (Retz.) Nees *ex* Wight & Arn., Edinburgh New Philos. J. 15: 380 (1833). *Pharus aristatus* Retz., Observ. Bot. 5: 23 (1789). An annual or perennial herb with ovate-oblong, ovate-lanceolate or lanceolate leaves. Inhabits lowlands, tanks, marshes, margins of paddy fields, often form floating masses. Fl. & Fr.: 10–2. Seeds are useful in biliousness. Voucher: Sunzid 311 (DUSH).
**22. *Oplismenus burmanni*** (Retz.) P. Beauv., Ess. Agrostogr.: 54 (1812). *Panicum burmanni* Retz., Observ. Bot. 3: 10 (1783). An annual, prostrate or trailing, slender herb with lanceolate to narrowly ovate leaves. Grows in shady places, roadsides, moist places, village thickets and homestead areas. Fl. & Fr.: 9–1. Used as fodder. Voucher: Sunzid 331 (DUSH).
**23. *Paspalum notatum*** Flüggé, Gram. Monogr., Paspalum: 106 (1810). *Paspalum taphrophyllum* Steud., Syn. Pl. Glumac. 1: 19 (1853). A perennial herb with linear to lanceolate leaves. Grows in fallow lands, agricultural fields, roadsides and rarely village thickets. Fl. & Fr.: 10–4. Widely used as a forage crop for livestock. Voucher: Sunzid 335 (DUSH).

#### Family: Menispermaceae

**24. *Tinospora cordifolia*** (Willd.) Hook. f. & Thomson, Fl. Ind. 1: 184 (1855). *Menispermum cordifolium* Willd., Sp. Pl. 4: 826 (1806). A perennial vine with cordate, alternate leaves. Grows in scrub jungles and homestead areas. Fl. & Fr.: 5–10. Used for treatment of diabetes, cardiac diseases and rheumatism. Voucher: Sunzid 355 (DUSH).

#### Family: Ranunculaceae

**25. *Nigella sativa*** L., Sp. Pl.: 584 (1753). *Nigella cretica* Mill., Gard. Dict. 8(4): (1768). A slender annual herb with decompound leaves. Grows in agricultural lands, sometimes roadsides and homestead areas, also cultivated. Fl. & Fr.: 1–12. Seeds are used to treat gastro-intestinal disorders. Voucher: Sunzid 328 (DUSH).

#### Family: Vitaceae

**26. *Leea indica*** (Burm. f.) Merr., Philipp. J. Sci. 14: 245 (1919). *Staphylea indica* Burm. f., Fl. Ind.: 75 (1768). A shrub with oblong or elliptic-oblong leaves. Grows in village thickets, scrub jungles, homestead areas and rarely in roadsides. Fl. & Fr.: 7–1. Leaves, roots and stem are used to treat inflammation, skin disease and gastro-intestinal disorders. Voucher: Sunzid 318 (DUSH).

#### Family: Fabaceae

**27. *Butea monosperma*** (Lam.) Kuntze, Revis. Gen. Pl.1: 202 (1891). *Erythrina monosperma* Lam., Encycl. 1: 391 (1785). A deciduous, perennial tree with trifoliate leaves. Inhabits roadsides, scrublands and sometimes in homestead areas. Fl. & Fr.: 2–4. Seeds are used to treat skin disease, piles and urinary tract disorders. Voucher: Sunzid 369 (DUSH).
**28. *Crotalaria juncea*** L., Sp. Pl.: 714 (1753). *Crotalaria benghalensis* Lam., Encycl. 2: 196 (1786). An erect annual herb with simple, oblong to elliptic-lanceolate leaves. Grows in roadsides, banks of ponds, homestead areas and rarely in scrub jungles. Fl. & Fr.: 4–9. Paste of fresh root is applied externally to treat snakebite. Voucher: Sunzid 370 (DUSH).
**29. *Desmodium triflorum*** (L.) DC., Prodr. 2: 334 (1825). *Nicolsonia triflora* (L.) Griseb., Abh. Königl. Ges. Wiss. Göttingen 7: 202 (1857). A prostrate to erect perennial herb with obovate, trifoliate leaves. Grows in open fields, pastures, disturbed areas and open woodlands. Fl. & Fr.: 6–10. Plant extract is used as laxative and often to treat bone fracture. Voucher: Sunzid 371 (DUSH).
**30. *Erythrina variegata*** L., Herb. Amb.: 122 (1754). *Erythrina indica* Lam., Encycl. 2: 391 (1786). A small to medium sized deciduous perennial tree with orbicular trifoliate leaves. Inhabits in village thickets, swamp and homestead areas. Fl. & Fr.: 2–5. Leaves are used to treat inflammation and pain. Voucher: Sunzid 305 (DUSH).

#### Family: Moraceae

**31. *Ficus benjamina*** L., Mant. Pl.: 129 (1767). *Urostigma benjaminum* (L.) Miq., London J. Bot. 6: 583 (1847). An evergreen tree with ovate to ovate-lanceolate leaves. Found in open places, roadsides and jungles. Fl. & Fr.: 7–11. Latex and fruit extracts are used to treat piles and skin diseases. Voucher: Sunzid 376 (DUSH).
**32. *Ficus racemosa*** L., Sp. Pl.: 1060 (1753). *Ficus goolereea* Roxb., Fl. Ind. ed. 3: 538 (1832). A large tree, with ovate-elliptic to oblong-elliptic leaves. Grows on riparian areas, open woodlands and sometimes in homestead areas. Fl. & Fr.: 3–5 & 9–11. Fruits are used to treat diarrhea. Latex is administered to treat hemorrhoids. Root sap is utilized to alleviate problems of diabetes. Voucher: Sunzid 377 (DUSH).
**33. *Ficus rumphii*** Blume, Bijdr. Fl. Ned. Ind.: 437 (1825). *Urostigma rumphii* (Blume) Miq., Syst. Verz. Ind. Archip. 2: 90 (1854). A small, perennial tree, with obovate to obovate-cordate leaves. Inhabits scrub jungles, roadsides and rarely in homestead areas. Fl. & Fr.: 3–11. Fruits are edible, leaf and bark juice is used to treat asthma. Voucher: Sunzid 378 (DUSH).

#### Family: Urticaceae

**34. *Dendrocnide sinuata*** (Blume) Chew, Gard. Bull. Singapore 21: 206 (1965). *Urtica sinuata* Blume, Bijdr. Fl. Ned. Ind.: 605 (1826). A dioecious shrub or small tree with ovate to lanceolate, caducous leaves. Grows on village thickets, roadsides, moist shady places and sometimes homestead areas. Fl. & Fr.: 6–2. Bark is occasionally used to make ropes. Sap is used as hair wash. Voucher: Sunzid 296 (DUSH).
**35. *Pouzolzia zeylanica*** (L.) Benn., Pl. Jav. Rar.: 67 (1838). *Parietaria zeylanica* L., Sp. Pl.: 1052 (1753). A monoecious perennial herb with ovate or elliptic-ovate leaves. Grows in roadsides, fallow land, near wetlands, shady places and homestead areas. Fl. & Fr.: 6–12. Poultice of leaves is used as a vermifuge. Voucher: Sunzid 343 (DUSH).

#### Family: Cucurbitaceae

**36. *Cucurbita moschata*** Duchesne, Ess. Hist. Nat. Courges : 7 (1786). *Cucurbita hippopera* Ser., Fl. Jard. 2: 531 (1847). An annual climbing herb with broadly ovate to ovate-orbicular leaves. Grows in open and sunny places and homestead areas. Fl. & Fr.: 3–12. Leaves and flowers are used as vegetables. Decoction of stem is used to treat diabetes. Voucher: Sunzid 293 (DUSH).
**37. *Trichosanthes cucumerina*** L., Sp. Pl.: 1008 (1753). *Anguina cucumerina* (L.) Kuntze, Revis. Gen. Pl. 1: 254 (1891). An annual climbing herb with orbicular-reniform or broadly ovate leaves. Inhabits homestead areas and open places, also found in forest thickets. Fl. & Fr.: 6–10. Decoction of roots is used as anthelmintic. Voucher: Sunzid 358 (DUSH).

#### Family: Oxalidaceae

**38. *Oxalis articulata*** Savigny, Encycl. 4: 686 (1798). *Acetosella articulata* (Savigny) Kuntze, Revis. Gen. Pl. 1: 91 (1891). A perennial, procumbent herb with palmately trifoliate leaves. Frequently grows on the paddy field, near wetlands, moist and shady places. Fl. & Fr.: 9–5. Leaves are consumed as vegetable. Voucher: Sunzid 333 (DUSH).

#### Family: Passifloraceae

**39. *Adenia trilobata*** (Roxb.) Engl., Bot. Jahrb. Syst. 14: 375 (1891). *Modecca trilobata* Roxb., Pl. Coromandel. 3: 93 (1820). A perennial climber with cordate, deeply 3-lobed leaves. Inhabits scrub jungles and rarely roadsides and homestead areas. Fl. & Fr.: 10–12. Decoction of leaves is used to treat neurological disorders. Voucher: Sunzid 281 (DUSH).

#### Family: Euphorbiaceae

**40. *Euphorbia hirta*** L., Sp. Pl. 1: 454 (1753). *Desmonema hirta* Raf., Atlantic J. 1: 178 (1833). A prostrate, annual herb with elliptic, hairy leaves. Inhabits roadsides, fallow lands, paddy fields, homestead areas and riverbanks. Fl. & Fr.: 7–12. Different plant parts are used to treat a wide variety of disorders including respiratory illness, digestive tract disorders, jaundice and gonorrhea. Voucher: Sunzid 367 (DUSH).
**41. *Euphorbia tithymaloides*** L., Sp. Pl.: 453 (1753). *Tithymalus tithymaloides* (L.) Croizat, Amer. J. Bot. 24: 704 (1937). A sub-succulent perennial undershrub with ovate to elliptic-ovate leaves. Inhabits in homestead areas, roadsides and shady places. Fl. & Fr.: 3–6. Milky latex is applied to warts and scorpion sting. The plant is also used as fence or hedge.: Voucher: Sunzid 306 (DUSH).
**42. *Jatropha gossypiifolia*** L., Sp. Pl. 2: 1006 (1753). *Adenoropium gossypiifolium* Pohl, Pl. Bras. Icon. Descr. 1: 16 (1826). A shrub with palmately lobed, alternate leaves. Grows in disturbed areas, gardens, fallow lands and sometimes in homestead areas. Fl. & Fr.: 4–8. Seeds and fruits are used against influenza and as a sedative, analgesic or anti-diarrheal agents. Voucher: Sunzid 368 (DUSH).
**43. *Mallotus philippensis*** (Lam.) Müll.Arg., Linnaea 34: 196 (1865). *Croton philippensis* Lam., Encycl. 2: 206 (1786). A small, evergreen perennial tree with ovate to ovate-lanceolate leaves. Inhabits in scrub jungle, rarely found in homestead areas. Fl. & Fr.: 1–12. Decoction of fruits is used in treating diabetes. Voucher: Sunzid 323 (DUSH).

#### Family: Combretaceae

**44. *Terminalia arjuna*** (Roxb. *ex* DC.) Wight & Arn., Prodr. Fl. Ind. Orient. 1: 314 (1834). *Pentaptera arjuna* Roxb. *ex* DC., Prodr. 3: 15 (1828). A medium to large tree with oblong to ovate-oblong leaves. Inhabits roadside, scrub jungles and open areas. Fl. & Fr.: 4–10. Used to treat cardiac problems and diabetes. Voucher: Sunzid 354 (DUSH).

#### Family: Lythraceae

**45. *Cuphea hyssopifolia*** Kunth, Nov. Gen. Sp. 6: 199 (1824). *Cuphea rivularis* Seem., Bot. Voy. Herald: 121 (1854). A small herb with lanceolate to linear-lanceolate leaves. Grows in the garden, homestead areas, sometimes along roadsides. Fl. & Fr.: 9–11. Valued for ornamental and aesthetic purposes. Voucher: Sunzid 294 (DUSH).
**46. *Lagerstroemia indica*** L., Syst. Nat., ed.10 (2): 1076 (1759). *Lagerstroemia chinensis* L., Amoen. Acad. 4: 137 (1759). A large shrub with elliptic, oblong, obovate or suborbicular leaves. Inhabits semi-shaded places and gardens. Fl. & Fr.: 6–11. Bark is considered as stimulant and febrifuge. Voucher: Sunzid 316 (DUSH).

#### Family: Onagraceae

**47. *Ludwigia hyssopifolia*** (G. Don) Exell, Garc. Ort. 5: 471 (1957). *Jussiaea hyssopifolia* G. Don, Gen. Hist. 2: 693 (1832). An erect, much-branched, perennial herb with lanceolate to oblong or elliptic leaves. Grows near wetlands, waste places, marshy fields, roadsides, fallow lands and bank of freshwater streams. Fl. & Fr.: 1–12. Whole plant parts are used in the treatment of diarrhea. Voucher: Sunzid 321 (DUSH).

#### Family: Myrtaceae

**48. *Syzygium cumini*** (L.) Skeels, Bull. Bur. Pl. Industr. 248: 25 (1912). *Syzygium fruticosum* DC., Prodr. 3: 260 (1828). An evergreen tree with elliptic, oblong to ovate leaves. Inhabits roadsides, homestead areas, scrub jungles and open fields. Fl. & Fr.: 3–6. Fruits are edible. Bark is used in the treatment of helminthiasis. Voucher: Sunzid 379 (DUSH).

#### Family: Rutaceae

**49. *Bergera koenigii*** L., Mant. Pl.: 565 (1767). *Murraya koenigii* (L.) Spreng., Syst. Veg., 16(2): 315 (1825). A large shrub or small tree with ovate to lanceolate leaves. Grows in village thickets, open places and homestead areas. Fl. & Fr.: 2–5. Leaves are used as a fixative in perfume and soap industry. Voucher: Sunzid 326 (DUSH).
**50. *Citrus maxima*** (Burm.) Merr., Interpr. Herb. Amboin.: 296 (1917). *Aurantium maximum* Burm., Auctuarium, Sign. Z, 1, Verso (1755). A small, evergreen tree with obcordate leaves. Observed in roadsides and homestead gardens. Fl. & Fr.: 2–11. Fruit juice is used to treat influenza and catarrh. Voucher: Sunzid 290 (DUSH).
**51. *Murraya paniculata*** (L.) Jack, Malayan Misc. 1(5): 31 (1820). *Murraya exotica* L., Mant. Pl. 2: 563 (1771). A perennial shrub or small tree with ovate, ovate-elliptic, oblong-elliptic to ovate leaves. Inhabits roadsides, gardens, scrub jungles, village thickets and homestead areas. Fl. & Fr.: 3–1. Leaves and stems are used to treat diarrhoea, dysentery and toothache. Voucher: Sunzid 327 (DUSH).

#### Family: Malvaceae

**52. *Melochia corchorifolia*** L., Sp. Pl.: 675 (1753). *Riedlea corchorifolia* (L.) DC., Prodr. 1: 491 (1824). An erect, annual herb with oval, round and slightly cuneate leaves. Inhabits roadsides, scrub jungles, homestead areas and open places. Fl. & Fr.: 3–6. Leaves are seeds are used to treat abdominal disorders. Voucher: Sunzid 375 (DUSH).
**53. *Microcos paniculata*** L., Sp. Pl.: 514 (1753). *Grewia microcos* L., Syst. Nat. 12 (2): 602 (1767). A perennial shrub with elliptic-lanceolate or ovate-lanceolate leaves. Grows in forest thickets, riverbanks and rarely in homestead areas. Fl. & Fr.: 8–4. Leaf extract is taken internally to treat jaundice and diarrhea. Fruits and barks have anti-inflammatory and analgesic properties. Voucher: Sunzid 381 (DUSH).
**54. *Sida acuta*** Burm. f., Fl. Indica: 147 (1768). *Sida crassa* Gand., Bull. Soc. Bot. France 71: 628 (1924). An annual woody herb with lanceolate to linear, elliptic-lanceolate or ovate-oblong leaves. Found in waste places, roadsides, dams, fields and fallow lands. Fl. & Fr.: 9–5. Stem produces good fibre. Leaves are used to treat testicular swellings and elephantiasis. Voucher: Sunzid 345 (DUSH).

#### Family: Cleomaceae

**55. *Cleome rutidosperma*** DC., Prodr. 1: 241 (1824). *Sieruela rutidosperma* (DC.) Roalson & J.C. Hall, Syst. Bot. 42: 938 (2017). An erect, annual herb with alternate, compound leaves. Inhabits fallow lands, paddy fields, homestead areas, roadsides and shady places. Fl. & Fr.: 5–12. Leaves, roots and seeds are used in the treatment of helminthiasis and diarrhoea. Voucher: Sunzid 322 (DUSH).
**56. *Cleome viscosa*** L., Sp. Pl. 2: 672 (1753). *Cleome acutifolia* Elmer, Leafl. Philipp. Bot. 7: 2574 (1915). An erect, annual herb with alternate, palmately compound leaves. Inhabits roadsides, fallow lands, dry and humid areas. Fl. & Fr.: 1–12. Whole plant extract is used to treat malarial fever, skin diseases and uterine complaints. Voucher: Sunzid 364 (DUSH).

#### Family: Polygonaceae

**57. *Persicaria lapathifolia*** (L.) Delarbre, Fl. Auvergne 2: 519 (1800). *Polygonum lapathifolium* L., Sp. Pl.: 360 (1753). An annual herb with lanceolate leaves. Grows in marshy areas, occasionally in cultivated fields. Fl. & Fr.: 10–11. Leaf extract is applied to treat skin sores, aerial plant parts possess anticancer properties. Voucher: Sunzid 336 (DUSH).
**58. *Persicaria orientalis*** (L.) Spach, Hist. Nat. Vég. 10: 537 (1841). *Polygonum orientale* L., Sp. Pl.: 362 (1753). An erect, annual herb with ovate, ovate-cordate to ovate-lanceolate leaves. Observed in marshy places, bank of rivers, ponds, canals, ditches and similar other water bodies. Fl. & Fr.: 3–8. Leaf extract is used as tonic and to treat helminthiasis. Fishermen use the plant to catch earthworms for angling. Voucher: Sunzid 380 (DUSH).

#### Family: Amaranthaceae

**59. *Celosia argentea*** L., Sp. Pl.: 205 (1753). *Celosia castrensis* L., Sp. Pl., ed. 2.: 297 (1762). An erect, annual herb with simple or ascending branches. Inhabits gardens and homestead areas. Fl. & Fr.: 9–1. Used as ornamental plant, leaves possess anti-diarrheal properties. Voucher: Sunzid 288 (DUSH).
**60. *Dysphania ambrosioides*** (L.) Mosyakin & Clemants, Ukrayins’k. Bot. Zhurn. 59(4): 382 (2002). *Chenopodium ambrosioides* L., Sp. Pl.: 219 (1753). An erect, annual herb with lanceolate to lanceolate-elliptical leaves. Inhabits open fields, roadsides, gardens and waste areas. Fl. & Fr.: 7–9. Seed extract is taken internally to treat problems of helminthiasis. Voucher: Sunzid 365 (DUSH).

#### Family: Portulacaceae

**61. *Portulaca grandiflora*** Hook., Bot. Mag. 56: 2885 (1829). *Portulaca pilosa* var. *grandiflora* (Hook.) Kuntze, Revis. Gen. Pl. 3(3): 15 (1898). An annual or perennial herb with succulent stems and leaves. Found in sandy alluvial soils, homestead areas, near wetlands, moist and shady places. Fl. & Fr.: 3–8. Valued for ornamental uses. Voucher: Sunzid 339 (DUSH).
**62. *Portulaca oleracea*** L., Sp. Pl.: 445 (1753). *Portulaca hortensis* Rupr., Fl. Ingrica: 388 (1854). An erect or prostrate annual herb with ovate-oblong to linear-oblong leaves. Grows along roadsides, agricultural fields and homestead areas. Fl. & Fr.: 5–8. Whole plant extract is used as diuretic, sedative, analgesic and tonic. Voucher: Sunzid 340 (DUSH).

#### Family: Cornaceae

**63. *Alangium chinense*** (Lour.) Harms, Ber. Deutsch. Bot. Ges. 15: 24 (1897). *Stylidium chinense* Lour., Fl. Cochinch.: 221 (1790). A small deciduous tree with ovate to ovate-cordate leaves. Inhabits scrub jungle, roadside and rarely homestead areas. Fl. & Fr.: 4–10. Foliage is used as fodder. Timber is utilized as fuel. Voucher: Sunzid 282 (DUSH).

#### Family: Balsaminaceae

**64. *Impatiens balsamina*** L., Sp. Pl.: 938 (1753). *Impatiens coccinea* Sims, Bot. Mag. 31: 1250 (1809). An erect annual herb with lanceolate-elliptic leaves. Inhabits open places, frequently near cultivated areas. Fl. & Fr.: 3–10. Decoction of leaves is taken internally to treat irregular menses. Voucher: Sunzid 313 (DUSH).

#### Family: Lecythidaceae

**65. *Careya arborea*** Roxb., Pl. Coromandel 3: 14 (1819). *Barringtonia arborea* (Roxb.) F. Muell., Fragm. 5: 184 (1866). A perennial, deciduous tree with broadly ovate, glabrous leaves. Observed in scrub jungles, roadsides and sometimes in homestead areas. Fl. & Fr.: 3–6. Used to treat diarrhoea, skin disease and fever. Voucher: Sunzid 373 (DUSH).

#### Family: Sapotaceae

**66. *Mimusops elengi*** L., Sp. Pl.: 349 (1753). *Kaukenia elengi* (L.) Kuntze, Revis. Gen. Pl. 2: 406 (1891). A large evergreen tree, with dense rounded crown and elliptic-oblong leaves. Grows in homestead areas, fields and open places. Fl. & Fr.: 3–6. Decoction of bark is used to treat bleeding gums. Voucher: Sunzid 324 (DUSH).

#### Family: Rubiaceae

**67. *Dentella repens*** (L.) J.R. Forst. & G. Forst., Char. Gen. Pl.: 26 (1776). *Oldenlandia repens* L., Mant. Pl. 1: 40 (1767). An annual prostrate herb with opposite, sessile or subsessile leaves. Frequently grows in wet places, shady places, and near wetlands. Fl. & Fr.: 12–7. Leaves and stem extracts are used in the treatment of diarrhea and constipation. Leaf paste is applied externally to treat problems of Eczema. Voucher: Sunzid 297 (DUSH).
**68. *Scleromitrion diffusum*** (Willd.) R.J. Wang, J. Trop. Subtrop. Bot. 22: 440 (2014). *Hedyotis diffusa* Willd., Sp. Pl., ed. 4. 1: 566 (1798). An annual, erect or semi-prostrate herb with linear to linear-lanceolate leaves. Inhabits near wetlands, moist and shady places and homestead areas. Fl. & Fr.: 7–12. Used as fodder. Voucher: Sunzid 310 (DUSH).
**69. *Spermacoce exilis*** (L.O. Williams) C.D. Adams ex W.C. Burger & C.M. Taylor, Fieldiana Bot. 33: 316 (1993). *Borreria repens* DC., Prodr. 4: 542 (1830). An annual, decumbent or procumbent herb with elliptic to elliptic-lanceolate leaves. Found along roadsides, plain land and forest periphery. Fl. & Fr.: 1–12. Utilized as fuel. Voucher: Sunzid 348 (DUSH).

#### Family: Convolvulaceae

**70. *Argyreia nervosa*** (Burm. f.) Bojer, Hortus Maurit.: 224 (1837). *Convolvulus nervosus* Burm. f., Fl. Ind.: 48 (1768). A perennial climbing vine with ovate to orbicular leaves. Found in open areas, along riverbanks and in disturbed or cultivated areas. Fl. & Fr.: 5–12. Leaves possess antidiabetic properties. Roots are used to treat neurological disorders. Voucher: Sunzid 283 (DUSH).
**71. *Evolvulus nummularius*** (L.) L., Sp. Pl., ed. 2.: 391 (1762). *Evolvulus repens* Parodi, Contr. Fl. Paraguay 1: 29 (1877). A prostrate, perennial herb with ovate to orbicular leaves. Inhabits in open and shady areas, scrub jungles, roadside and homestead areas. Fl. & Fr.: 1–12. Crushed plant parts are used to treat wounds. Voucher: Sunzid 307 (DUSH).
**72. *Ipomoea carnea*** Jacq., Enum. Syst. Pl.: 13 (1760). *Convolvulus carneus* (Jacq.) Spreng., Syst. Veg., ed. 16. 1: 602 (1824). A shrub with ovate to lanceolate leaves. Found in wetlands, moist places, along riverbanks and disturbed areas. Fl. & Fr.: 3–9. Fibre obtained from the species is used to make ropes and cordage. Voucher: Sunzid 314 (DUSH).

#### Family: Solanaceae

**73. *Solanum sisymbriifolium*** Lam., Tabl. Encycl. 2: 25 (1794). *Solanum rogersii* S. Moore, J. Bot. 58: 78 (1920). An annual or perennial, very prickly erect herb with sinuately lobed leaves. Inhabits rail-track, roadsides, village thickets and scrub jungles. Fl. & Fr.: 1–12. Leaves are used to treat diarrhoea and dysentery. Voucher: Sunzid 347 (DUSH).

#### Family: Oleaceae

**74. *Nyctanthes arbor-tristis*** L., Sp. Pl.: 6 (1753). *Scabrita scabra* L., Syst. Nat., 12(2): 115 (1767). A shrub or small tree with elliptic to ovate leaves. Grows in homestead areas, rarely found in scrub jungles. Fl. & Fr.: 8–9. Leaves and roots are used to treat problems of diabetes. Also used for aesthetic purposes. Voucher: Sunzid 329 (DUSH).

#### Family: Linderniaceae

**75. *Bonnaya antipoda*** (L.) Druce, Rep. Bot. Soc. Brit. Isles 3: 415 (1914). *Ruellia antipoda* L., Sp. Pl.: 635 (1753). A prostrate, perennial herb with oblong-lanceolate, oblong-oblanceolate or linear-lanceolate leaves. Inhabits moist and shady places, near ponds and sometimes in homestead areas. Fl. & Fr.: 8–10. Serves as a source of fuel. Voucher: Sunzid 374 (DUSH).
**76. *Bonnaya ruelloides*** (Colsm.) Spreng., Syst. Veg., ed. 16. 1: 41 (1824). *Gratiola ruelloides* Colsm., Prodr. Descr. Gratiol.: 12 (1793). An annual herb with oblong-elliptic, ovate-oblong or circular leaves. Grows in moist and shady places and homestead areas. Fl. & Fr.: 7–9. Used as fodder. Voucher: Sunzid 285 (DUSH).
**77. *Lindernia rotundifolia*** (L.) Alston, Handb. Fl. Ceylon 6: 214 (1931). *Gratiola rotundifolia* L., Mant. Pl. 2: 174 (1771). An erect, sometimes diffused perennial herb with ovate to orbicular leaves. Observed growing on agricultural land after harvesting, open meadows and marshy areas. Fl. & Fr.: 8–11. Often used as aquarium plant for aesthetic purposes. Voucher: Sunzid 319 (DUSH).
**78. *Torenia crustacea*** (L.) Cham. & Schltdl., Linnaea 2: 570 (1827). *Capraria crustacea* L., Mant. Pl. 1: 87 (1767). A prostrate, annual herb with linear to lanceolate leaves. Inhabits moist and shady places, sometimes roadsides and near wetlands. Fl. & Fr.: 8–11. Used as an ornamental plant. Voucher: Sunzid 356 (DUSH).

#### Family: Acanthaceae

**79. *Ecbolium ligustrinum*** (Vahl) Vollesen, Kew Bull. 44: 651 (1989). *Justicia ligustrina* Vahl, Enum. Pl. 1: 118 (1804). An erect shrub. Grows in shady places and gardens. Fl. & Fr.: 1–12. Used to treat gout, jaundice and rheumatism. Voucher: Sunzid 360 (DUSH).
**80. *Strobilanthes hirta*** (Vahl) Blume, Bijdr. Fl. Ned. Ind.: 797 (1826). *Ruellia hirta* Vahl, Symb. Bot. 3: 84 (1794). A small, prostrate, softly hirsute or villous herb. Inhabits moist land, higher elevations and arid climate. Fl. & Fr.: 1–7. Dried plant parts are used as fuel. Voucher: Sunzid 351 (DUSH).
**81. *Ruellia tuberosa*** L., Sp. Pl. 2: 635 (1753). *Cryphiacanthus barbadensis* Nees, Ind. Sem. (WROCL): 1841 (1841). An erect, perennial herb with oblong-ovate leaves. Inhabits homestead areas, roadsides, grasslands and in shady places. Fl. & Fr.: 6–8. Leaves are used to treat helminthiasis. Voucher: Sunzid 292 (DUSH).
**82. *Hygrophila auriculata*** (Schumach.) Heine, Kew Bull. 16(2): 172 (1962). *Barleria longifolia* L., Cent. Pl. II: 22 (1756). An erect, perennial, aquatic or semi-aquatic herb with lanceolate to linear-lanceolate leaves. Grows in wetlands, marshes, riverbanks and ponds. Fl. & Fr.: 10–4. Whole plant is used for treatment of jaundice, body ache and as aphrodisiac. Voucher: Sunzid 353 (DUSH).

#### Family: Bignoniaceae

**83. *Oroxylum indicum*** (L.) Kurz, Forest Fl. Burma 2: 237 (1877). *Bignonia indica* L., Sp. Pl.: 625 (1753). A small to medium sized deciduous tree, with bi- or tri-pinnately compound leaves. Grows in scrub jungles, roadsides and open areas. Fl. & Fr.: 6–3. Used to treat problems of diabetes. Voucher: Sunzid 332 (DUSH).

#### Family: Verbenaceae

**84. *Phyla nodiflora*** (L.) Greene, Pittonia 4: 46 (1899). *Verbena nodiflora* L., Sp. Pl.: 20 (1753). A tough, perennial, creeping herb with obovate leaves. Inhabits open wastelands in moist and damp soils, dry riverbeds, fallow lands, village thickets and poorly drained soil. Fl. & Fr.: 1–12. Decoction of root is used as aphrodisiac. Voucher: Sunzid 337 (DUSH).

#### Family: Lamiaceae

**85. *Callicarpa macrophylla*** Vahl, Symb. Bot. 3: 13 (1794). *Callicarpa incana* Roxb., Fl. Ind. 1: 407 (1820). An evergreen shrub with ovate-lanceolate to oblong-lanceolate leaves. Observed growing in forest thickets, open places and rarely in homestead areas. Fl. & Fr.: 6–2. Leaves and roots are used to treat diarrhoea, fever and rheumatism. Voucher: Sunzid 286 (DUSH).
**86. *Coleus scutellarioides*** (L.) Benth. in N. Wallich, Pl. Asiat. Rar. 2: 16 (1830). *Ocimum scutellarioides* L., Sp. Pl., ed. 2.: 833 (1763). An annual herb with ovate to lanceolate leaves. Grows in homestead areas, shady places, sometimes in open places and near wetlands. Fl. & Fr.: 5–9. Valued for ornamental uses. Voucher: Sunzid 291 (DUSH).
**87. *Gmelina arborea*** Roxb., Hort. Bengal.: 46 (1814). *Gmelina sinuata* Link, Enum. Hort. Berol. Alt. 2: 128 (1822). A large deciduous tree with broadly ovate decussate leaves. Inhabits scrub jungle, sometimes roadsides and rarely homestead areas. Fl. & Fr.: 2–7. Timber is used to build high-quality furniture. Leaves possess anthelmintic properties. Voucher: Sunzid 309 (DUSH).
**88. *Leucas lavandulifolia*** Sm., A. Res. Cycl. 20: 2 (1812). *Leucas linifolia* (Roth) Spreng., Syst. Veg., ed. 16. 2: 743 (1825). An erect, annual or short-lived perennial herb with oblong-linear, linear-lanceolate or linear leaves. Inhabits roadsides, fallow lands, riverbanks, homestead areas, scrub jungles and shady places. Fl. & Fr.: 7–10. Whole parts are used to treat fever, asthma, psoriasis, dermatitis and snake bites. Voucher: Sunzid 372 (DUSH).
**89. *Pogostemon auricularius*** (L.) Hassk., Tijdschr. Natuurl. Gesch. Physiol. 10: 127 (1843). *Mentha auricularia* L., Mant. Pl. 1: 81 (1767). A slender, erect, annual herb with elliptic to ovate leaves. Grows along the roadsides, riversides, homestead areas and agricultural lands. Fl. & Fr.: 9–1. Used to treat hysteria, diarrhoea and stomach ache. Voucher: Sunzid 338 (DUSH).

#### Family: Campanulaceae

**90. *Lobelia chinensis*** Lour., Fl. Cochinch.: 514 (1790). *Dortmanna chinensis* (Lour.) Kuntze, Revis. Gen. Pl. 2: 972 (1891). A prostrate annual herb with denticulate leaves. Grows in agricultural lands, roadsides, riversides and open areas. Fl. & Fr.: 1–12. Used for ornamental purposes. Voucher: Sunzid 320 (DUSH).

#### Family: Menyanthaceae

**91. *Nymphoides indica*** (L.) Kuntze, Revis. Gen. Pl. 2: 429 (1891). *Menyanthes indica* L., Sp. Pl.: 145 (1753). A floating annual herb with orbicular, deeply cordate leaves. Grows in the margins of tanks, lakes, pools, rivers and wetland habitats. Fl. & Fr.: 10–2. Whole plant extract is used in the treatment of fever and jaundice. Voucher: Sunzid 330 (DUSH).

#### Family: Asteraceae

**92. *Acmella oleracea*** (L.) R.K. Jansen, Syst. Bot. Monogr. 8: 65 (1985). *Spilanthes oleracea* L., Syst. Nat., ed. 12. 2: 534 (1767). An erect perennial herb with triangular-ovate leaves. Grows in shady places, roadsides, riversides and homestead areas. Fl. & Fr.: 2–10. Boiled leaves are often taken internally to alleviate pain. Voucher: Sunzid 280 (DUSH).
**93. *Cyanthillium patulum*** (Aiton) H. Rob., Proc. Biol. Soc. Washington 103: 252 (1990). *Conyza patula* Aiton, Hort. Kew. 3: 184 (1789). An erect, rarely decumbent, annual herb. Inhabits waste places, shady and open homestead areas, scrub jungles and roadsides. Fl. & Fr.: 1–12. Used to feed animals. Leaves are used as vegetable. Voucher: Sunzid 359 (DUSH).
**94. *Eclipta prostrata*** (L.) L., Mant. Pl. 2: 286 (1771). *Verbesina prostrata* L., Sp. Pl.: 902 (1753). An erect annual herb with elliptic-lanceolate to ovate or obovate leaves. Grows in homestead areas, paddy field, scrub jungles and roadsides. Fl. & Fr.: 1–12. Used in the treatment of jaundice. Voucher: Sunzid 300 (DUSH).
**95. *Grangea maderaspatana*** (L.) Poir., Encycl. [J. Lamarck & al.] Suppl. 2. 825 (1812). *Artemisia maderaspatana* L., Sp. Pl.: 849 (1753). An erect, annual herb with linear to lanceolate leaves. Observed on roadsides, homestead areas, fallow lands, banks of ponds and shady places. Fl. & Fr.: 3–7. Plant extract is used to treat rheumatoid arthritis. Voucher: Sunzid 362 (DUSH).
**96. *Helichrysum luteoalbum*** (L.) Rchb., Handb. Gewächsk., ed. 2(2): 1460 (1829). *Gnaphalium luteoalbum* L., Sp. Pl.: 851 (1753). A prostrate to erect, annual herb with oblong to lanceolate leaves. Observed on meadows, fallow lands and disturbed areas. Fl. & Fr.: 3–4. Leaves are used as astringent, diuretic and febrifuge. Voucher: Sunzid 363 (DUSH).
**97. *Parthenium hysterophorus*** L., Sp. Pl.: 988 (1753). *Parthenium pinnatifidum* Stokes, Bot. Mat. Med. 4: 278 (1812). An annual herb with deeply lobed and pinnately divided leaves. Grows in disturbed areas, agricultural fields and open spaces. Fl. & Fr.: 11–3. Utilized as fuel. Voucher: Sunzid 334 (DUSH).
**98. *Sphagneticola trilobata*** (L.) Pruski, Mem. New York Bot. Gard. 78: 114 (1996). *Silphium trilobatum* L., Syst. Nat., 10(2): 1233 (1759). A perennial creeper with three-lobed leaves. Observed in roadsides, gardens, natural and semi-natural areas. Fl. & Fr.: 3–8. Mainly used for ornamental purposes. Voucher: Sunzid 349 (DUSH).
**99. *Tridax procumbens*** L., Sp. Pl. 2: 900 (1753). *Balbisia elongata* Willd., Sp. Pl. 3: 2214 (1803). A prostrate, annual herb with oblong to ovate leaves. Inhabits open field, roadsides and waste areas. Fl. & Fr.: 6–9. Leaf extracts are taken internally for treating diarrhea. Voucher: Sunzid 361 (DUSH).

#### Family: Apiaceae

**100. *Eryngium foetidum*** L., Sp. Pl.: 232 (1753). *Eryngium antihystericum* Rottler, Acta Lit. Univ. Hafn. 1: 288 (1778). A prostrate herb with much branched stem. Grows on roadsides, moist places and steam banks. Fl. & Fr.: 4–1. Leaves are consumed as vegetables. Voucher: Sunzid 304 (DUSH).

### 3.2 Drug design endeavor

#### 3.2.1 Site-specific molecular docking

**Fig 3:**
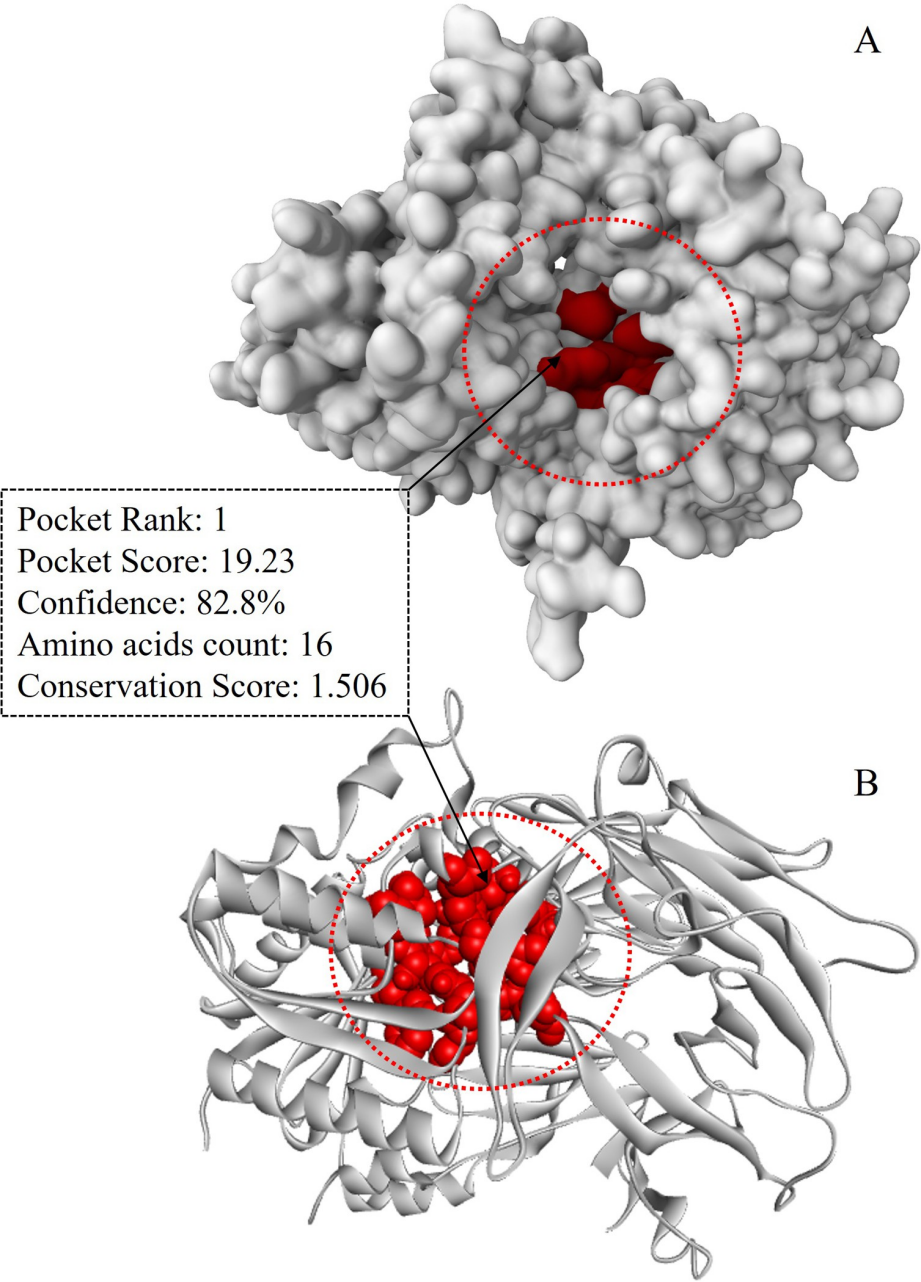
Binding cavity identification. A. Surface view showing active site inside the red circle, B. Ribbon view indicating active site inside the red circle.

**Fig 4:**
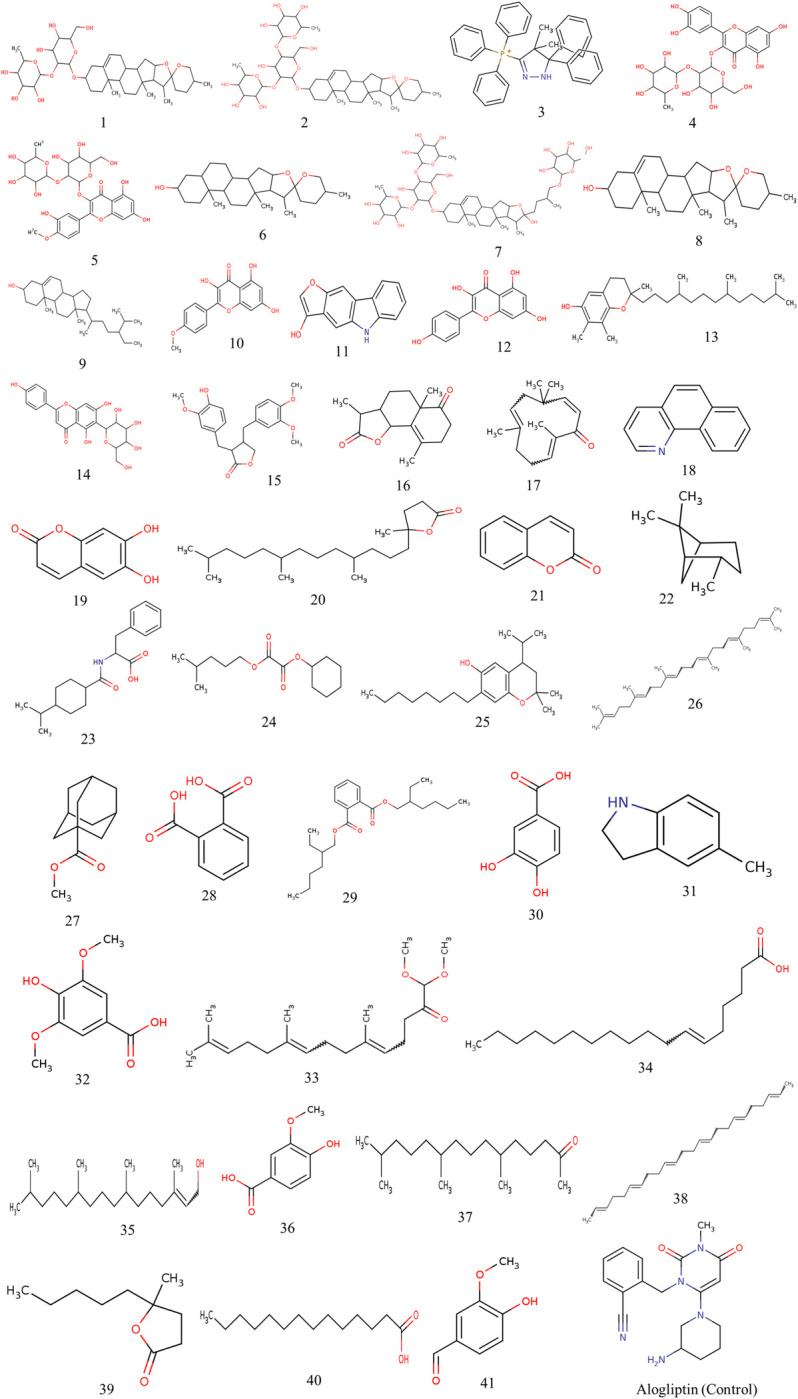
Two dimensional structures of all the phytochemicals and control drug Alogliptin used for molecular docking analysis. The number of compounds is in accordance with the serial number enlisted in Table 1.

The PrankWeb server predicted eight active sites, and the best-ranked site was selected based on pocket and confidence scores. The selected active site demonstrated the highest pocket score (19.23) with a confidence level of 82.8% and encompassed a total of 16 amino acids as binding site residues (Fig 3). The binding site residues were Arg125, Glu205, Glu206, Phe357, Tyr547, Cys551, Lys554, Trp629, Ser630, Tyr631, Val656, Tyr662, Tyr666, Asn710, His740 and Gly741. Molecular docking analysis was successfully conducted for 41 phytochemicals of *C*. *cuspidatus* where the binding affinity varied from -5.3 to -10.1 kcal/mol. The highest binding affinity (-10.1 kcal/mol) was recorded for Prosapogenin A, while the lowest (-5.3 kcal/mol) was noted for 4-hydroxy-3-methoxybenzaldehyde (Table 1). Alogliptin scored -7.0 kcal/mol and based on that 53.66% compounds were eliminated and 46.34% compounds were accepted for ADMET analysis. Following ADMET analysis, Tigogenin and Diosgenin emerged as the most promising lead compounds, scoring -9.0 kcal/mol and -8.5 kcal/mol, respectively. Two dimensional structures of all the ligands have been visualized in Fig 4.

**Table 1:** Binding affinity of phytochemicals after molecular docking analysis targeting DPP4 protein.

Sl. No.	Phytochemicals	PubChem CID	Chemical formula	Binding affinity (kcal/mol)
1	Prosapogenin A	11061578	C_39_H_62_O_12_	-10.1
2	Dioscin	119245	C_45_H_72_O_16_	-9.6
3	(4,4-dimethyl-5,5-diphenyl-1H-pyrazol-3-yl)-triphenylphosphanium	4051786	C_35_H_32_N_2_P^+^	-9.2
4	Quercetin 3-O-neohesperidoside	5491657	C_27_H_30_O_16_	-9.2
5	Tamarixetin 3-neohesperidoside	44258037	C_28_H_32_O_16_	-9.1
6	**Tigogenin**	99516	C_27_H_44_O_3_	-9
7	Protodioscin	441891	C_51_H_84_O_22_	-8.7
8	**Diosgenin**	99474	C_27_H_42_O_3_	-8.5
9	Beta-sitosterol	222284	C_29_H_50_O	-8.4
10	Kaempferide	5281666	C_16_H_12_O_6 _	-8.1
11	3-hydroxy-1h-benzo[b]furo[2,3-f]indole	129780852	C_14_H_9_NO_2_	-7.8
12	Kaempferol	5280863	C_15_H_10_O_6_	-7.8
13	Gamma-Tocopherol	92729	C_28_H_48_O_2_	-7.7
14	Isovitexin	162350	C_21_H_20_O_10_	-7.7
15	Arctigenin	64981	C_21_H_24_O_6 _	-7.6
16	Taurin	211707	C_15_H_20_O_3_	-7.3
17	Zerumbone	5470187	C_15_H_22_O	-7.3
18	Benzo[h]quinoline	9191	C_13_H_9_N	-7.2
19	Esculetin	5281416	C_9_H_6_O_4_	-7.2
20	4,8,12,16-Tetramethylheptadecan-4-olide	567149	C_21_H_40_O_2_	-6.7
21	Coumarin	323	C_9_H_6_O_2_	-6.7
22	Pinane	12314300	C_10_H_18_	-6.7
23	Nateglinide	5311309	C_19_H_27_NO_3_	-6.6
24	Oxalic acid	6421306	C_14_H_24_O_4_	-6.6
25	2,2-dimethyl-7-octyl-4-propan-2-yl-3,4-dihydrochromen-6-ol	91221	C_22_H_36_O_2_	-6.5
26	Squalene	638072	C_30_H_50_	-6.5
27	Methyl adamantane-1-carboxylate	136553	C_12_H_18_O_2_	-6.4
28	Phthalic acid	1017	C_8_H_6_O_4_	-6.4
29	Bis(2-ethylhexyl) phthalate	8343	C_24_H_38_O_4_	-6.3
30	Protocatechuic acid	72	C_7_H_6_O_4_	-6.3
31	5-methyl-2,3-dihydro-1H-indole	14023679	C_9_H_11_N	-6.1
32	4-hydroxy-3,5-dimethoxybenzoic acid	10742	C_9_H_10_O_5_	-6
33	1,1-dimethoxy-6,10,14-trimethylpentadeca-5,9,13-trien-2-one	53946054	C_20_H_34_O_3_	-5.9
34	Petroselaidic acid	5282754	C_18_H_34_O_2_	-5.9
35	Phytol	5280435	C_20_H_40_O	-5.9
36	Vanillic acid	8468	C_8_H_8_O_4_	-5.9
37	6,10,14-trimethylpentadecan-2-one	10408	C_18_H_36_O	-5.7
38	2,6,10,14,18,22-Tetracosahexaene	57417215	C_24_H_38_	-5.5
39	5-methyl-5-pentyloxolan-2-one	103702	C_10_H_18_O_2_	-5.5
40	Tetradecanoic acid	11005	C_14_H_28_O_2_	-5.4
41	4-hydroxy-3-methoxybenzaldehyde	1183	C_8_H_8_O_3_	-5.3
	Alogliptin (control)	11450633	C_18_H_21_N_5_O_2 _	-7

#### 3.2.2 Molecular interaction analysis

**Fig 5:**
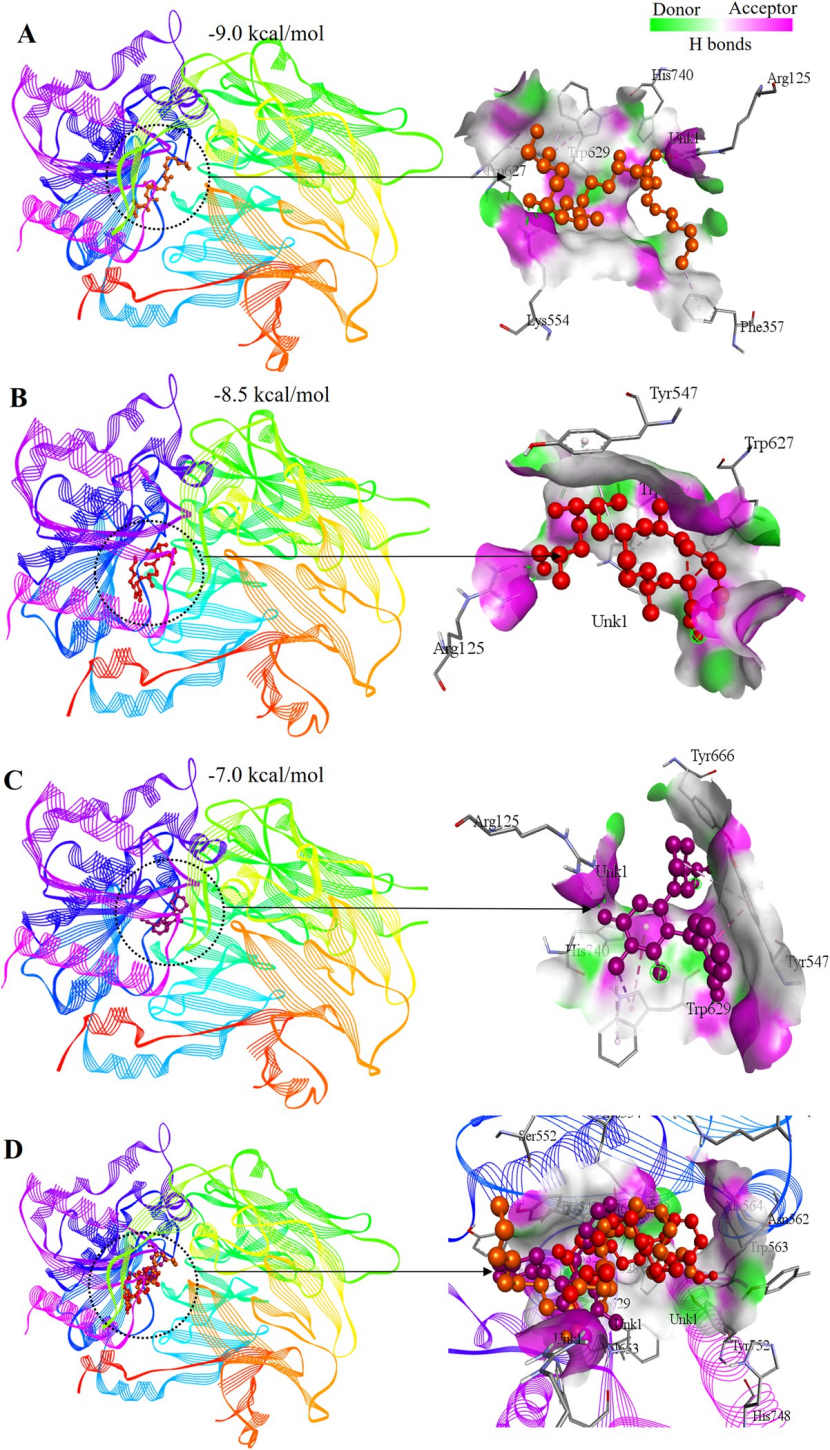
Docked complexes with three dimensional molecular interactions between the lead compounds and target protein. A. Interaction of Tigogenin. B. Interaction of Diosgenin. C. Interaction of Alogliptin. D. The two lead candidates and control are superimposed at the active site of DPP4 protein.

**Fig 6:**
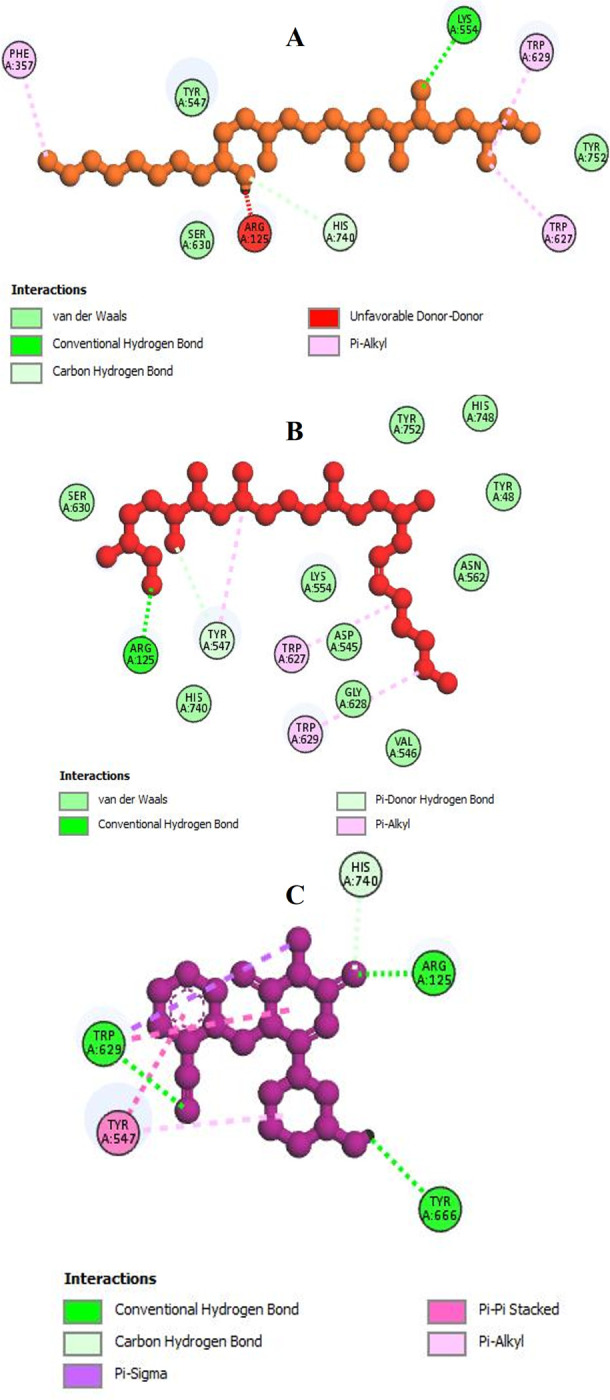
Two dimensional molecular interactions between the ligands and target protein showing hydrogen bonding and hydrophobic interactions. A. Tigogenin. B. Diosgenin. C. Alogliptin.

3D visualization of the docked complexes depicted site-specific binding of the best two lead compounds and the control in the active site of the target protein (Fig 5). Protein-ligand interactions unveiled both hydrophobic interactions and conventional hydrogen bonds (Fig 6 and Table 2). Tigogenin interacted with Arg125, Phe357, Lys554, Trp627, Trp629 and His740, forming two conventional hydrogen bonds only with Lys554 (Fig 6A). Diosgenin interacted with Arg125, Tyr547, Trp627 and Trp629 residues, with Arg125 involved in conventional hydrogen bonding (Fig 6B). The control drug Alogliptin displayed very similar interacting patterns where it formed interactions with Arg125, Tyr547, Trp629, Tyr666 and His740 residues. Conventional hydrogen bonding was observed in the residues Arg125, Trp629 and Tyr666 (Fig 6C).

**Table 2:** Interacting residues of the two lead compounds along with the control drug Alogliptin.

Proteins	Ligands	Interacting sites	Residues in conventional hydrogen bonds (distance in Å)	Number of conventional hydrogen bonds	Residues in hydrophobic bonds (distance in Å)	Binding affinity (kcal/mol)
DPP4 (2ONC)	Tigogenin	Arg125, Phe357, Lys554, Trp627, Trp629, His740	Lys554^(2.72, 2.81)^	2	Arg125^(1.04)^, Phe357^(4.19)^, Trp627^(4.82)^, Trp629^(4.92, 5.27)^, His740^(3.50)^	-9.0
	Diosgenin	Arg125, Tyr547, Trp627, Trp629	Arg125^(2.13, 2.34)^	2	Tyr547^(4.10, 5.40)^, Trp627^(5.49)^, Trp629^(5.15)^	-8.5
	Alogliptin	Arg125, Tyr547, Trp629, Tyr666, His740	Arg125^(2.20, 2.21)^, Trp629^(2.46)^, Tyr666^(2.76)^	4	Tyr547^(4.20, 4.94)^, Trp629^(3.99, 4.93, 5.60)^ His740^(3.59)^	-7.0

#### 3.2.3 Drug candidacy evaluation via ADMET analysis

Nineteen phytochemicals scored higher than the control drug in molecular docking analysis and were subsequently subjected to ADMET analysis to evaluated their drug-likeness properties. ADMET analysis revealed Tigogenin and Diosgenin to have the best pharmacokinetic, pharmacodynamic and toxicity properties (Table 3). Tigogenin exhibited a slightly higher molecular weight (416.64 g/mol) than Diosgenin (414.62 g/mol). Molecular weight of Alogliptin was lower (339.39 g/mol) than that of both lead compounds. H-bond donating and accepting parameters were very similar for the leads and the control. Molar refractivity was recorded as lower in the control drug compared to both lead compounds. Topological Polar Surface Area (TPSA) was recorded same for Diosgenin and Tigogenin (38.69 Å^2^), whereas for the control it was slightly higher (97.05 Å^2^). Both Diosgenin and Tigogenin were found to be more lipophilic than Alogliptin. Both the lead compounds and the control exhibited higher gastrointestinal (GI) absorption, and none of the compounds demonstrated inhibitory properties against CYP isoforms, yielding results comparable to the control. Diosgenin was found to be more soluble in water than Tigogenin. In Lipinski’s parameters, both Diosgenin and Tigogenin exhibited drug-likeness, each with only one violation. However, when assessed against the Veber and Egan criteria, their drug candidacy was affirmed without any violations. The bioavailability scores were identical for both lead compounds and Alogliptin. Similar to Alogliptin, both Diosgenin and Tigogenin exhibited zero alerts in PAINS test. The synthetic accessibility scores for the two lead compounds were moderate (Table 3).

**Table 3:** ADMET properties of the proposed compounds and Alogliptin.

Parameters		Tigogenin	Diosgenin	Alogliptin (control)
Physicochemical properties	Formula	C_27_H_44_O_3_	C_27_H_42_O_3_	C_18_H_21_N_5_O_2_
	Molecular weight	416.64 g/mol	414.62 g/mol	339.39 g/mol
	H-bond acceptor	3	3	4
	H-bond donors	1	1	1
	Molar refractivity	122.07	121.59	98.84
	TPSA	38.69 Å^2^	38.69 Å^2^	97.05 Å^2^
Lipophilicity	Consensus Log *P*_*o/w*_	5.22	5.01	0.90
Pharmacokinetics	GI absorption	High	High	High
	CYP isoforms (CYP1A2, 2C19, 2C9, 2D6, 3A4)inhibitor	No	No	No
	P-gp substrate	No	No	No
	Log *K*_p_ (skin permeation)	-4.80 cm/s	-4.23 cm/s	Log *K*_p_ (skin permeation)
Water Solubility	Log *S* (SILICOS-IT)	-4.49	-4.51	-3.31
	Solubility	1.34e-02 mg/ml; 3.22e-05 mol/l	1.29e-02 mg/ml; 3.10e-05 mol/l	1.65e-01 mg/ml; 4.85e-04 mol/l
	Class	Moderately soluble	Moderately soluble	Soluble
Druglikeness	Lipinski	Yes; 1 Violations: MLOGP > 4.15	Yes; 1 Violation: MLOGP > 4.15	Yes
Medicinal Chemistry	PAINS	0 alert	0 alert	0 alert
	Synthetic accessibility	6.88	6.94	3.51
Toxicity	Hepatotoxicity	Non-toxic	Non-toxic	Non-toxic
	Carcinogenicity	Non-toxic	Non-toxic	Non-toxic
	Mutagenicity	Non-toxic	Non-toxic	Non-toxic
	Cytotoxicity	Non-toxic	Non-toxic	Non-toxic
	Acute oral toxicity	Non-toxic	Non-toxic	Toxic
	Eye irritation and corrosion	Non-toxic	Non-toxic	Toxic

In the toxicity test, Diosgenin and Tigogenin outperformed the control drug Alogliptin, as evidenced by the results presented in Table 3. Alogliptin exhibited toxicity in the acute oral toxicity criterion, whereas both lead compounds showed no toxic effects. Likewise, Alogliptin demonstrated toxic effects in the eye irritation and corrosion criterion, while the two leads showed no signs of toxicity in this parameter. In all other toxicity parameters, the two leads showcased profiles similar to the control drug (Table 3).

#### 3.2.4 Molecular dynamics simulation (MDS)

**Fig 7:**
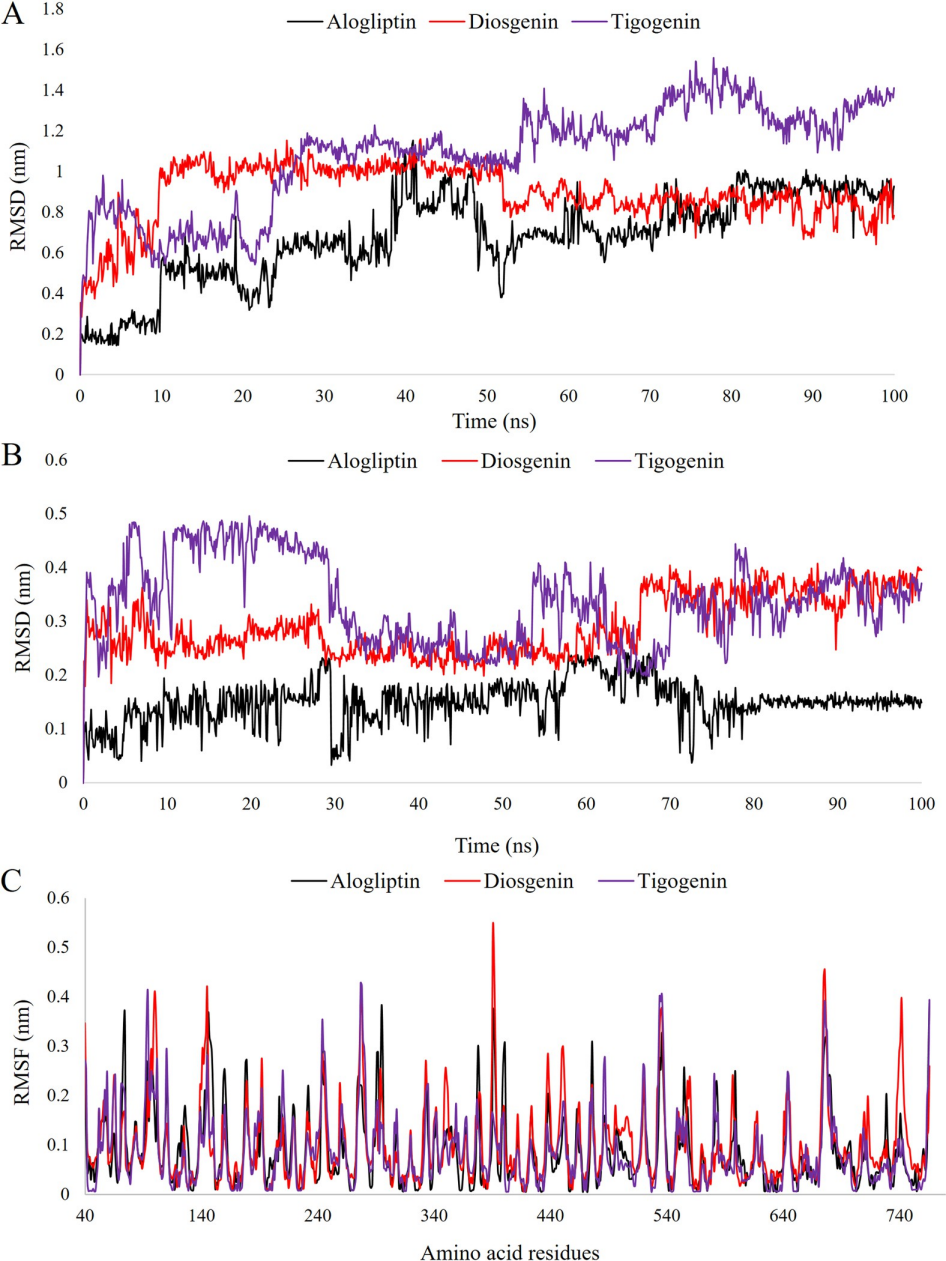
Simulation trajectory of the lead candidates and control drug for 100 ns. A. RMSD focusing on backbone after least squares fitting to backbone. B. RMSD focusing on the ligand after least square fitting to protein. C. RMSF analysis.

**Fig 8:**
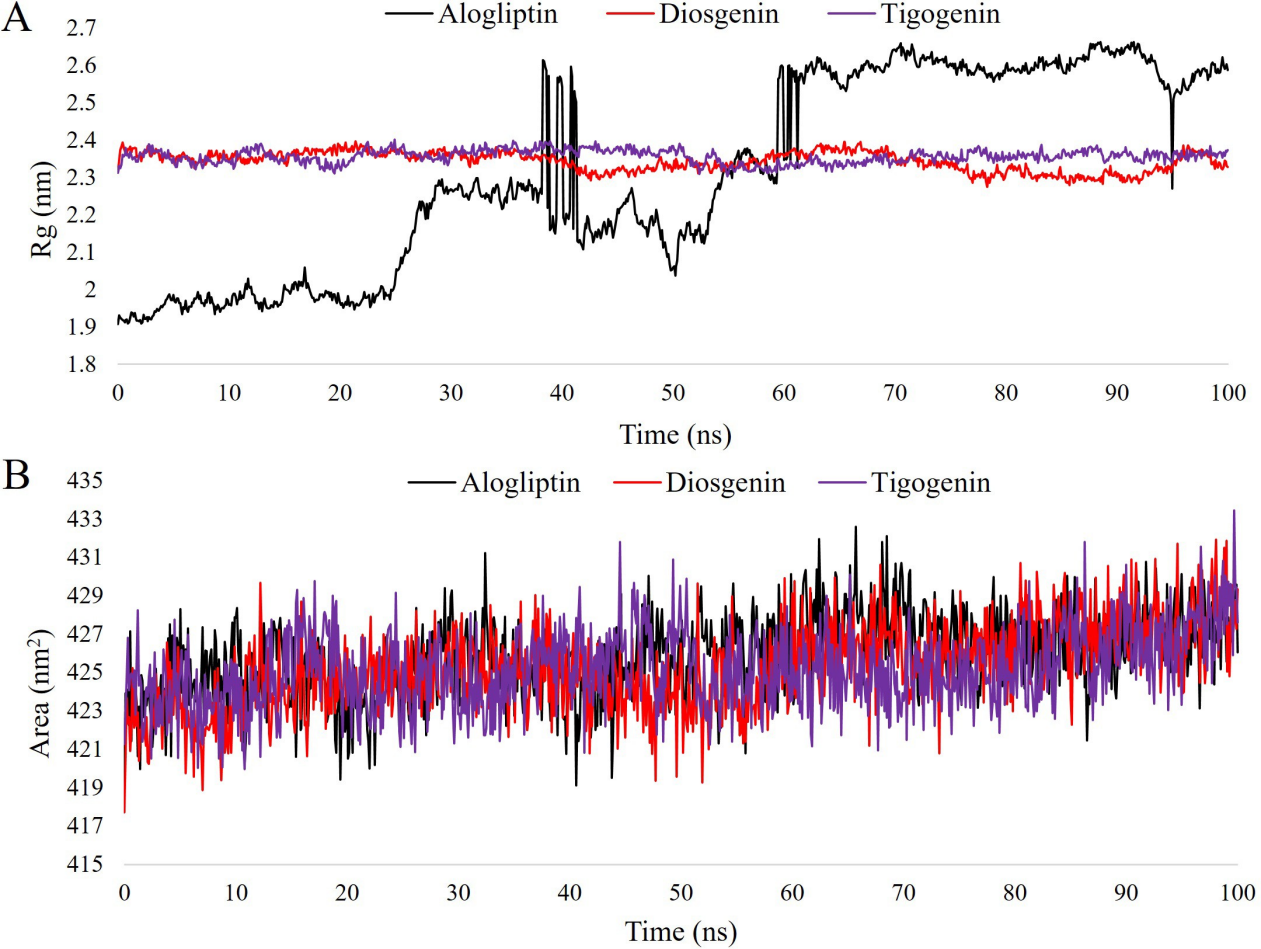
Simulation trajectory of the lead candidates and control drug for 100 ns. A. Rg (total) analysis. B. SASA (total) analysis.

The two leads demonstrated dynamic behavior closely paralleled to that of the control drug Alogliptin (Figs 7 and 8). RMSD (Root Mean Square Deviation) analysis, focusing on the backbone after least squares fitting to backbone, revealed that Diosgenin closely tracked Alogliptin from 55 to 100 ns (Fig 7A). Tigogenin showed a close similarity with Diosgenin and Alogliptin between 40 to 48 ns. After 54 ns, Tigogenin displayed a slight elevation and continued up to 100 ns with no drastic fluctuation. The mean RMSD was recorded highest for Tigogenin (1.08 nm), followed by Diosgenin (0.89 nm) and Alogliptin (0.67 nm).

RMSD analysis, with a focus on the ligand after least square fitting to the protein, showcased a close affinity between the leads and the control from 30 to 55 ns (Fig 7B). Beyond the 70 ns mark, Diosgenin and Tigogenin closely mirrored each other, attaining stability. Despite a slightly increased RMSD, both compounds maintained a consistent trajectory, aligning with the control drug and exhibiting minimal fluctuations after the 70 ns time point. The mean RMSD of Alogliptin, Diosgenin and Tigogenin were recorded as 0.14, 0.39 and 0.37 nm, respectively.

RMSF (Root Mean Square Fluctuation) study presented very similar regional flexibility profiles for the lead compounds and the control drug (Fig 7C). In the binding cavity, mean RMSF values were recorded as 0.06, 0.03 and 0.08 nm for Alogliptin, Diosgenin and Tigogenin, respectively. Diosgenin exhibited better results compared to both the control and Tigogenin. Among the five interacting residues of Alogliptin, RMSF values were recorded as 0.154 (Arg125), 0.040 (Tyr547), 0.006 (Trp629), 0.039 (Tyr666) and 0.091 (His740). Similarly, for Diosgenin, RMSF values were 0.078 (Arg125), 0.052 (Tyr547), 0.005 (Trp627) and 0.005 (Trp629). For Tigogenin, RMSF values were 0.139 (Arg125), 0.084 (Phe357), 0.158 (Lys554), 0.024 (Trp627), 0.025 (Trp629) and 0.111 (His740).

Rg (Radius of Gyration) study showcased very similar behavior for Diosgenin and Tigogenin (Fig 8A) with mean Rg values of 2.34 and 2.35 nm, respectively. Alogliptin showed initial fluctuations, and after 62 ns, it stabilized, maintaining a steady trajectory until 93 ns. At the 94^th^ ns, there was a reduction in Rg from 2.53 to 2.31 nm and became stabilized again to 2.52 nm at the 95^th^ ns. After 95 ns, the trajectory remained consistent up to 100 ns.

**Fig 9:**
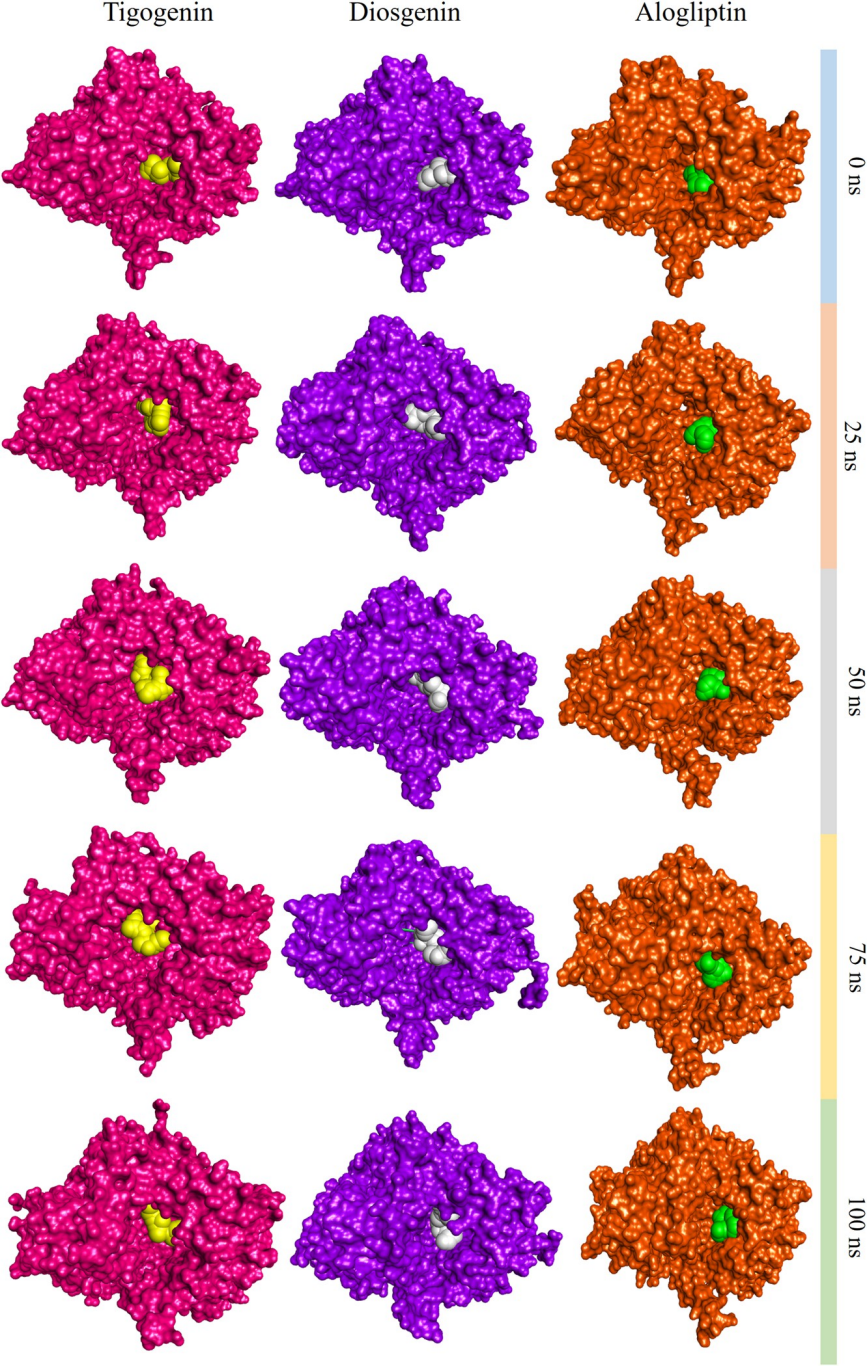
Snapshots of the simulation trajectory at regular intervals showing stability and compactness of Tigogenin, Diosgenin and Alogliptin.

SASA (Solvent Accessible Surface Area) analysis revealed very similar results for the two lead compounds and the control drug. Mean SASA values were recorded as 426.06, 429.35 and 427.61 nm^2^ for Alogliptin, Diosgenin and Tigogenin, respectively (Fig 8B). Both Diosgenin and Tigogenin followed Alogliptin in close proximity throughout the entire simulation trajectory up to 100 ns. Simulation snapshots supported the structural stability and compactness of the lead compounds after binding with the target protein (Fig 9).

#### 3.2.5 Principal Component Analysis (PCA)

**Fig 10:**
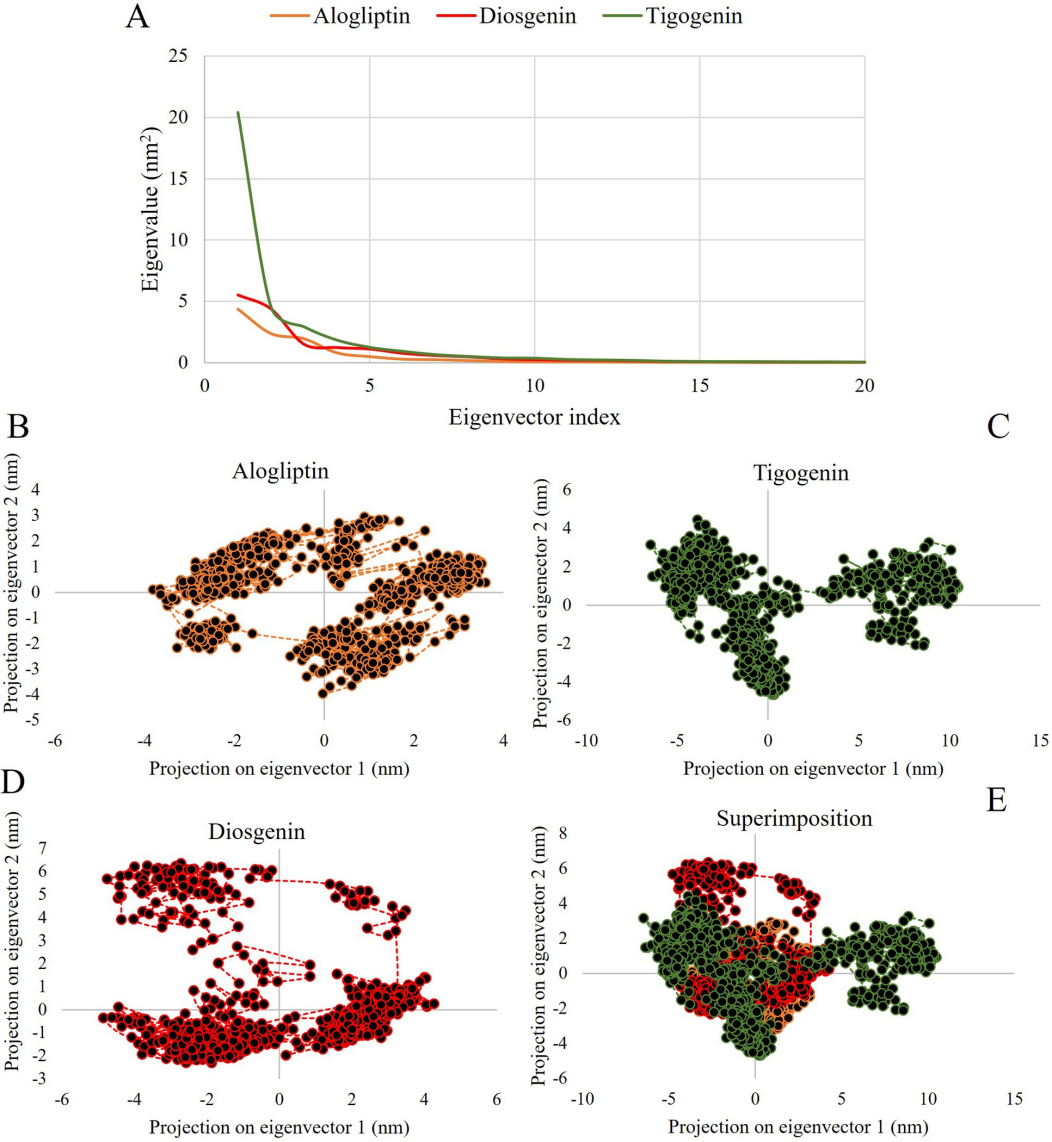
Principle component analysis showing eigenvalue versus eigenvector index and projection of the motion in phase space for the lead compounds and control drug.

PCA was conducted to explore significant motions during ligand binding in the top two lead-protein complexes and the control-protein complex. Eigenvalues were computed for the initial 20 eigenvectors, with average values of 0.55 nm^2^, 0.84 nm^2^, and 1.76 nm^2^ for Alogliptin, Diosgenin, and Tigogenin, respectively (Fig 10A). Lower trace values of the covariance matrix indicated decreased flexibility and overall stability. The phase space characteristics of the complexes aligned with Fig 10A, where a complex occupying less phase space and forming a stable cluster represented greater stability. Conversely, a complex occupying more space and forming a non-stable cluster indicated lesser stability. The Alogliptin-complex occupied the least phase space, followed by the Diosgenin-complex and Tigogenin-complex. This outcome underscores the superior stability of Diosgenin over Tigogenin compared to the control drug Alogliptin (Fig 10B–10E).

#### 3.2.6 Gibbs free energy landscape analysis

**Fig 11:**
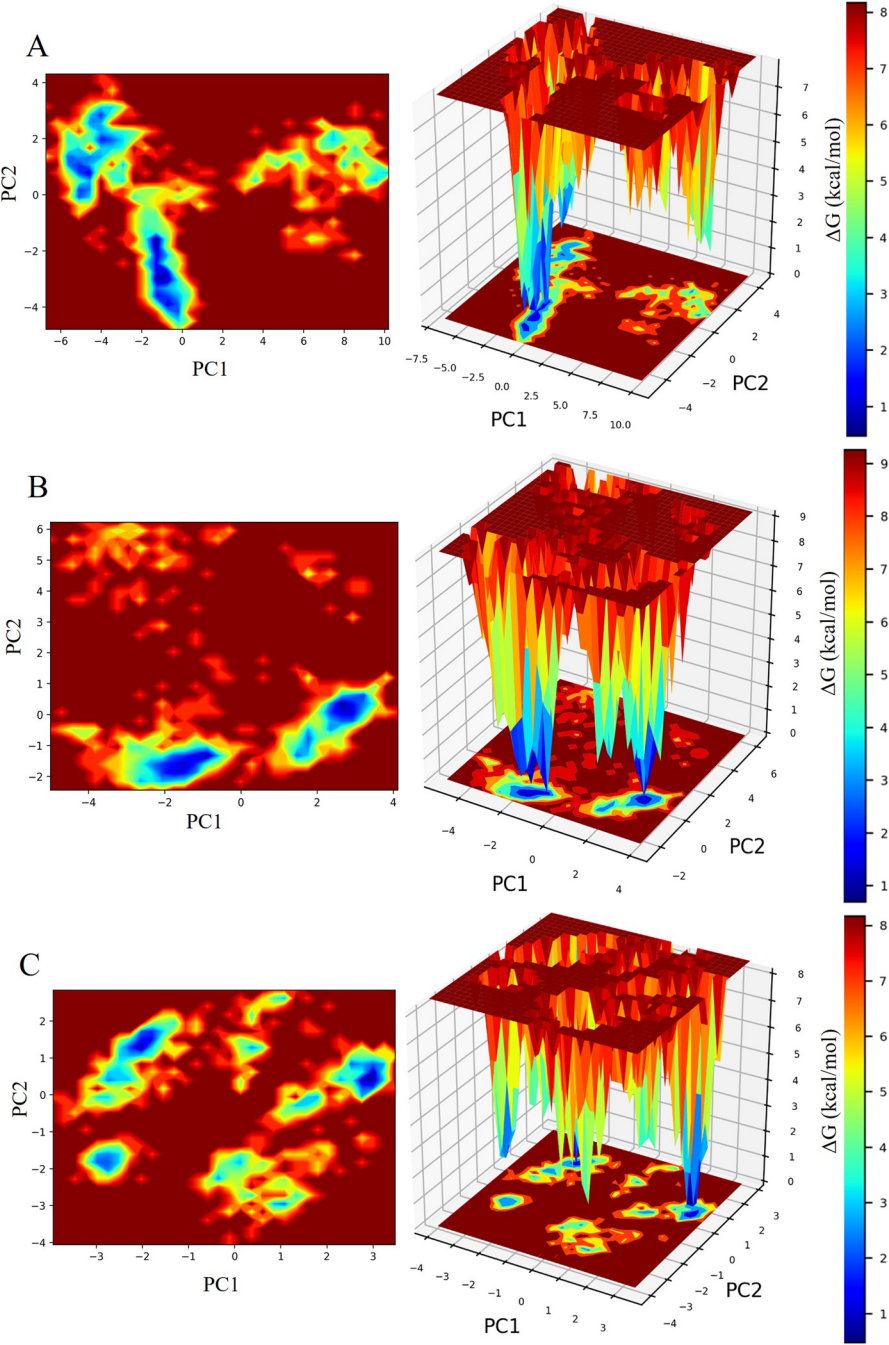
Two- and three-dimensional representation of Gibbs free energy landscapes. Deeper blue color represents lower energy systems. A. FEL of Tigogenin. B. FEL of Diosgenin. C. FEL of Alogliptin.

FEL (Free energy landscape) study unveiled no single energy minimum for all the three complexes and exhibited multiple energy minima (Fig 11). All the complexes achieved minimum energy corresponding to their most stable conformations. Smaller and more centralized blue areas depicted greater stability in the complexes. Both Diosgenin and Tigogenin exhibited profile very similar to that of Alogliptin. However, between the two lead compounds, Diosgenin displayed smaller and centralized blue areas than Tigogenin, indicating the superior potential of Diosgenin to induce the target protein to enter a local energy-minimal state.

#### 3.2.7 Free binding energy

The MM/GBSA approach revealed the highest score for Diosgenin (-47.36 kcal/mol) followed by Tigogenin (-46.70 kcal/mol) and Alogliptin (-46.32 kcal/mol) (Table 4). The higher free binding energy of the lead compounds further corroborated the docking protocol. The superior free binding energy of Diosgenin and Tigogenin indicated their favorable energetic behavior over Alogliptin.

**Table 4:** Estimation of free binding energies of Tigogenin, Diosgenin and Alogliptin.

Complexes	Δ G Bind (kcal/mol)	Δ G Coulomb (kcal/mol)	Δ G Covalent (kcal/mol)	Δ G Lipo (kcal/mol)	Δ G Bind Solv GB (kcal/mol)	Δ G Bind vdW (kcal/mol)
Tigogenin-DPP4 complex	-46.70	-9.85	4.83	-36.37	34.51	-38.84
Diosgenin-DPP4 complex	-47.36	-10.78	5.26	-37.24	34.54	-38.13
Alogliptin-DPP4 complex (control)	-46.32	-20.43	2.67	-25.89	33.98	-33.61

#### 3.2.8 Structural analogs and drug target class

**Fig 12:**
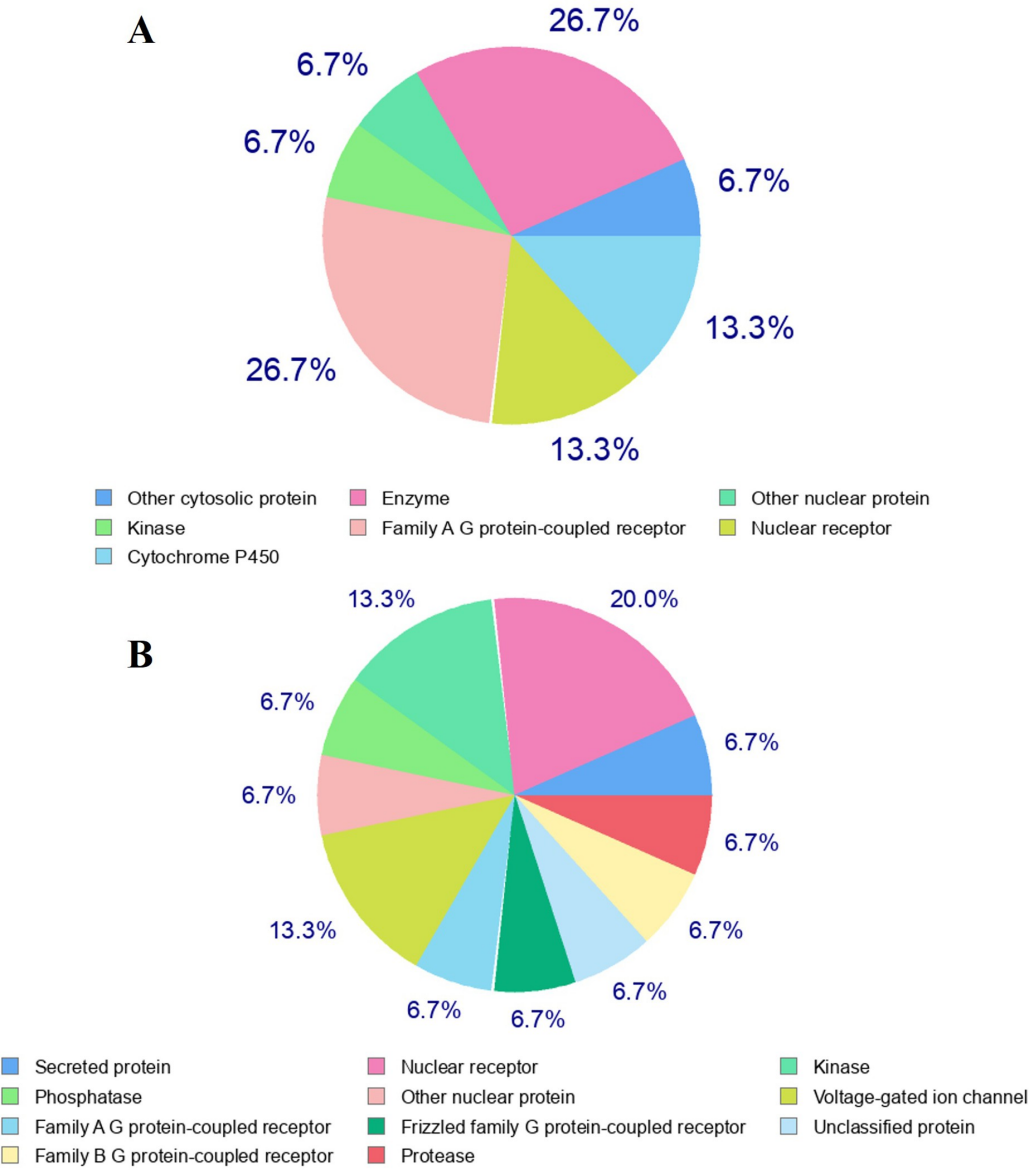
Predicted drug target classes of the lead candidates focusing on *Homo sapiens* dataset. A. Tigogenin. B. Diosgenin.

Tigogenin was predicted to interact with 7 distinct drug target classes, as illustrated in Fig 12A. Enzymes emerged as the most prevalent target class, constituting 26.7% of the predictions. In the case of Diosgenin, predictions spanned 11 different target classes, with nuclear receptors being the most frequently predicted class, accounting for 20% of the interactions (Fig 12B).

The prediction of structurally similar analogs unveiled both experimental and investigational analogs. Smilagenin, 2-Hexyloxy-6-Hydroxymethyl-Tetrahydro-Pyran-3 and Lauryl glucoside were predicted as analogs of Tigogenin with confidence levels of 100%, 73% and 70%, respectively. Similarly, Ruscogenin, Caprospinol and Smilagenin were predicted as analogous to Diosgenin with confidence levels of 98%, 78% and 68%, respectively (Table 5).

**Table 5:** Structurally similar analogs of Tigogenin and Diosgenin.

Phytocompounds	Analogs	Drug Bank ID	Confidence	Stage
Tigogenin	i. Smilagenin	DB05450	100%	Investigational
	ii. 2-Hexyloxy-6-Hydroxymethyl-Tetrahydro-Pyran-3	DB03772	73%	Experimental
	iii. Lauryl glucoside	DB14746	70%	Experimental
Diosgenin	i. Ruscogenin	DB15830	98%	Experimental
	ii. Caprospinol	DB05263	78%	Investigational
	iii. Smilagenin	DB05450	68%	Investigational

## 4. Discussion

The present investigation documented 100 additional taxa of angiosperms from Gafargaon subdistrict. Among the documented taxa, Magnoliopsida (dicots) were dominant, and the majority of the species were herbs. This finding is consistent with similar other studies where additional taxa of angiosperms were uncovered [52–54]. Citation frequency was highest for *C*. *cuspidatus* regarding its use in treating diabetes; therefore, this species was selected for a drug design endeavor. The selection of this species based on ethnobotanical perception aligns with several other studies where phytochemicals from specific taxa were subjected for drug design based on their ethnomedicinal significance [6, 55, 56]. *C*. *cuspidatus* has been reported to possess antidiabetic properties in various studies. Kaur and Mannan (2021) conducted an *in vitro* investigation using the Bovine Serum Albumin (BSA)-Glucose Assay and found that, after 15 days of incubation, *C*. *cuspidatus* extract significantly reduced fluorescence intensity in a concentration-dependent manner. This suggests that the plant extract inhibited the formation of advanced glycation end-products, potentially providing protection against diabetic complications [57]. Deogade et al. (2017) performed an *in vivo* study using *C*. *cuspidatus* leaf extract in diabetic rabbits to assess its anti-hyperglycemic activity. The study revealed a notable decrease in blood glucose levels, indicating the antidiabetic potential of *C*. *cuspidatus* in an *in vivo* setting [58]. In the present investigation, we employed an *in silico* strategy to validate previous *in vitro* and *in vivo* findings of antidiabetic efficacy of *C*. *cuspidatus* targeting the DPP4 protein. Alogliptin, an FDA-approved DPP4 inhibitor was used as the control drug for virtual screening of phytocompounds through molecular docking analysis. Alogliptin improves glucose control in diabetes by prolonging the action of incretin hormone. Additionally, Alogliptin boosts insulin secretion, reduces glucagon production, and lowers blood sugar levels after meals [31]. The virtual screening revealed that 46.34% of phytocompounds scored higher than Alogliptin (-7.0 kcal/mol) with binding affinity ranging from -7.2 to -10.1 kcal/mol. Chigurupati et al. (2022) conducted molecular docking employing PyRx 0.8 software to assess antidiabetic efficacy of *Moringa oleifera* phytocompounds targeting the human pancreatic alpha-amylase (HPA) protein (PDB ID: 5EOF). The study demonstrated a binding affinity ranging from -6.0 to -9.4 kcal/mol for the investigated phytochemicals of *M*. *olerifera* [59]. In our study, both the upper and lower thresholds of the binding affinity of the top selected compounds were higher, providing justification for the antidiabetic efficacy of *C*. *cuspidatus* phytocompounds from an energetic perspective. Molecular interaction analysis revealed that the best two lead compounds, Tigogenin and Diosgenin, interacted with more than two active site residues (Table 2). Both the lead compounds and the control drug showed interaction with Arg125 and Trp629. This suggests that these two residues are likely catalytically important sites, serving as potential drug surface hotspots and playing a significant role in the drug’s mechanism of action. ADMET analysis revealed the drug-likeness of Tigogenin and Diosgenin, and our result was found to be consistent with previous studies [11, 60]. Lipinski parameters are essential in the development of potential lead candidates. Tigogenin and Diosgenin demonstrated favorable outcomes in Lipinski parameters, consistent with findings from previous SBDD investigation [11, 61]. Forecasting CYP isoform inhibition stands as a pivotal stage in ADMET investigations. This assessment is crucial for gauging the potential outcomes of drug candidates, confirming their safety and therapeutic efficacy [61]. In the present study, Tigogenin and Diosgenin displayed CYP inhibition tendency very similar to Alogliptin. In toxicity parameter, these two lead compounds showed better results than the control drug, further justifying their selection as potential inhibitors of DPP4. The PAINS (Pan-assay interference compounds) test identifies Pan-Assay Interference Compounds which can lead to deceptive results in drug screening assays. These compounds often exhibit reactive or promiscuous behavior, interfering with multiple targets and yielding false positive outcomes. By recognizing and filtering out such compounds early in the drug discovery process, this test enhances the efficiency and reliability of screening efforts, saving valuable time and resources [8]. In the current study, all lead candidates demonstrated zero alerts in PAINS assay, affirming their selection as the potential inhibitors against the DPP4 receptor. Molecular Dynamic Simulation unveiled noteworthy stability and compactness of Tigogenin and Diosgenin when compared to Alogliptin (Figs 7 and 8). RMSD, RMSF, Rg and SASA profiles of the two leads closely resembled those of Alogliptin. The similar dynamic behavior of the two leads corroborated their selection as potential antidiabetic drug candidates. Simulation trajectories were consistent with findings from previous studies using GROMACS software [13, 60]. PCA, also referred to as covariance analysis or essential dynamics (ED), is a systematic approach for delineating the collective and comprehensive movements within a protein system by methodically reducing the complexity of the system [62]. PCA analysis showcased similar dynamic motion for both the leads and the control. However, among the two leads, Diosgenin was found to be more stable than Tigogenin (Fig 10). The results from Gibbs Free Energy Landscape (FEL) supported the findings of PCA, indicating better stability of Diosgenin over Tigogenin (Fig 11). Both the leads and the control drug exhibited multiple energy minima, suggesting the existence of multiple stable states or configurations within the systems. The FEL and PCA analyses unraveled that the binding of Tigogenin and Diosgenin not only modified the conformation of DPP4 protein but also altered the essential dynamics for inhibition. The PCA and FEL assessments were consistent with findings from other studies [63, 64]. The MM/GBSA free binding energy was found to be higher in Diosgenin and Tigogenin than that of Alogliptin, which corroborated the docking protocol by eliminating the possibility of false-positive results. This implies that Diosgenin and Tigogenin are likely to form strong and stabilizing interactions with DPP4 upon binding, potentially leading to a favorable pharmacological response. Ahmed et al. (2022) proposed three antidiabetic lead compounds from *Piper betle* phytochemicals targeting alpha-amylase and alpha-glucosidase proteins. In that study, MM/GBSA free binding energy, estimated using Prime module of Schrödinger, ranged from -34.66 to -45.02 kcal/mol for the three leads against alpha-amylase protein. In the case of alpha-glucosidase, the binding energy spanned from -28.68 to -38.28 kcal/mol for the same three lead compounds of *P*. *betle* [65]. In our study, the binding energy varied from -46.70 to -47.36 kcal/mol, showing better results compared to the *P*. *betle* phytocompounds (Table 5). The prediction of drug targets may assist in identifying novel targets for Diosgenin and Tigogenin, while forecasting structurally analogous compounds streamlines the drug design process targeting the DPP4 protein [20].

The current study employed the SBDD approach to identify and validate potential inhibitors against the DPP4 protein; however, certain limitations are associated with this method. The computer-based tools used in the present study are valuable, though not always perfectly accurate. Consequently, the precise efficacy of Tigogenin and Diosgenin in hindering the target protein *in vitro* and *in vivo* remains uncertain, requiring further wet-lab validations. Additionally, SBDD methods encounter challenges in various aspects, including benchmarking, constrained prediction approaches, and a lack of diverse datasets for different computational analyses [66]. Despite these challenges, the current investigation has the potential to shed light on the role of *C*. *cuspidatus* in uncovering antidiabetic therapeutics.

## 5. Conclusion

In the present investigation, an interdisciplinary approach was employed to document additional angiosperm taxa in Gafargaon subdistrict and to guide drug design process from the floristics survey. The taxonomic inventory unveiled a total of 100 additional taxa of angiosperms under 90 genera and 46 families. Documenting the presence of these additional taxa will enrich the flora of Gafargaon and contribute to a deeper understanding of angiosperm diversity in this subdistrict. This would illuminate key aspects of conservation planning by identifying and safeguarding the plant species within Gafargaon, aiding in the formulation of targeted conservation strategies. Documentation of additional taxa also holds potential medicinal importance, uncovering new plant resources that could be explored for pharmaceutical applications and highlighting the interconnectedness of biodiversity, human health, and sustainable resource utilization. Ethnobotanical knowledge regarding the antidiabetic use of *C*. *cuspidatus* has been validated in computational framework employing a comprehensive SBDD protocol. Tigogenin and Diosgenin, identified as potential inhibitors against the DPP4 protein, demonstrated favorable pharmacokinetic and pharmacodynamic properties with no major side effects. These compounds showed noteworthy structural stability in a 100 ns simulation, and the MM/GBSA approach further corroborated their selection. The proposed lead candidates, Tigogenin and Diosgenin unveiled from the SBDD study hold promise for the development of novel inhibitors targeting the DPP4 protein to combat diabetes. In future, the efficacy of the lead candidates will be investigated through multiple *in vitro* enzymatic assays, encompassing the Dipeptidyl peptidase-4 inhibition assay, Alpha-amylase inhibition assay, and Aldose reductase inhibition assay. Favorable outcomes will guide subsequent *in vivo* investigations using animal models. Additionally, long-term endeavors will focus on fostering extensive collaborative research for development of novel antidiabetic therapeutics.

## Supporting information

S1 File Python scripts for generating 2D and 3D figures of Gibbs free energy landscape analysis. (DOCX)
