# Supplementary material for: From Flora to Pharmaceuticals: 100 new additions to angiosperms of Gafargaon subdistrict in Bangladesh and unraveling antidiabetic drug candidates targeting DPP4 through in silico approach
Source: PLoS One. 2024 Mar 29;19(3):e0301348. doi: 10.1371/journal.pone.0301348 (PMC10980240; doi:10.1371/journal.pone.0301348)
Supplement: S1 File — (DOCX) [file pone.0301348.s001.docx]

**Manuscript Title: “From Flora to Pharmaceuticals: 100 new additions to angiosperms of Gafargaon subdistrict in Bangladesh and unraveling antidiabetic drug candidates targeting DPP4 through *in silico* approach”**

**Python script for generating Figures of Gibbs free energy landscape**

**For 2D contour map**

import numpy as np

import matplotlib

# Set the backend to Agg before importing matplotlib.pyplot

matplotlib.use('Agg')

import matplotlib.pyplot as plt

from matplotlib import cm

from scipy.interpolate import griddata

from numpy import linspace

# Input file with x, y, z coordinates

x, y, z = np.loadtxt('gibbs.txt').T

X, Y = np.unique(x), np.unique(y)

xi = linspace(min(X), max(X), len(X))

yi = linspace(min(Y), max(Y), len(Y))

xi, yi = np.meshgrid(xi, yi)

X1, Y1 = np.meshgrid(X, Y)

Z = griddata((x, y), z, (X1, Y1), method='cubic')

# Create a figure

fig = plt.figure()

# Create a contour plot

cset = plt.contourf(X1, Y1, Z, cmap=cm.jet, antialiased=3, vmin=0, vmax=18)

# Set labels and tick sizes

plt.xlabel('PC1', fontsize=12)

plt.tick_params(axis="x", labelsize=6)

plt.ylabel('PC2', fontsize=12)

plt.tick_params(axis="y", labelsize=6)

# Save the plot as a PNG file

plt.savefig('2D_plot.png', dpi=300, bbox_inches='tight')

# Close the figure to prevent displaying it

plt.close()

# Optional: You can display a message indicating that the plot has been saved

print("2D plot saved as 2D_plot.png")

**For 3D map**

import numpy as np

import matplotlib

# Set the backend to Agg before importing matplotlib.pyplot

matplotlib.use('Agg')

import matplotlib.pyplot as plt

from mpl_toolkits.mplot3d import Axes3D

from matplotlib import cm

from scipy.interpolate import griddata

from numpy import linspace

# Input file with x, y, z coordinates

x, y, z = np.loadtxt('gibbs.txt').T

X, Y = np.unique(x), np.unique(y)

xi = linspace(min(X), max(X), len(X))

yi = linspace(min(Y), max(Y), len(Y))

xi, yi = np.meshgrid(xi, yi)

X1, Y1 = np.meshgrid(X, Y)

Z = griddata((x, y), z, (X1, Y1), method='cubic')

# Create a figure

fig = plt.figure()

# Create a 3D surface plot

ax = fig.add_subplot(111, projection='3d')

surf = ax.plot_surface(X1, Y1, Z, rstride=1, cstride=1, alpha=1, cmap=cm.jet, linewidth=0.0, antialiased=3)

cset = ax.contourf(X1, Y1, Z, zdir='z', offset=0, cmap=cm.jet, antialiased=3, vmin=0, vmax=7.8)

# Set labels and tick sizes

ax.set_xlabel('PC1', fontsize=12)

ax.tick_params(axis="x", labelsize=6)

ax.set_ylabel('PC2', fontsize=12)

ax.tick_params(axis="y", labelsize=6)

ax.set_zlabel('Z axis', fontsize=12)

ax.tick_params(axis="z", labelsize=6)

ax.set_zlim(0, 7.8)

# Add a color bar

cbar = fig.colorbar(cset, shrink=0.8, aspect=20, extend='neither', ticks=range(0, 21, 2))

# Save the plot as a PNG file

plt.savefig('3D_plot.png', dpi=300, bbox_inches='tight')

# Close the figure to prevent displaying it

plt.close()

# Optional: You can display a message indicating that the plot has been saved

print("3D plot saved as 3D_plot.png")
